# Oxidative Stress and Redox Imbalance in *Acanthamoeba* Keratitis: Host–Parasite Crosstalk and Therapeutic Perspectives

**DOI:** 10.3390/ijms27167293

**Published:** 2026-08-15

**Authors:** Roland Wesołowski, Paweł Sutkowy, Hanna Lesiewska, Anna Kieszkowska, Alina Woźniak

**Affiliations:** 1 Department of Medical Biology and Biochemistry, Faculty of Medicine, Ludwik Rydygier Collegium Medicum in Bydgoszcz, Nicolaus Copernicus University in Toruń, 85-092 Bydgoszcz, Poland; p.sutkowy@cm.umk.pl; 2 Department of Ophthalmology, Faculty of Medicine, Ludwik Rydygier Collegium Medicum in Bydgoszcz, Nicolaus Copernicus University in Toruń, 85-094 Bydgoszcz, Poland; hanna.lesiewska@cm.umk.pl; 3 Student Research Club of Medical Parasitology, Faculty of Medicine, Ludwik Rydygier Collegium Medicum in Bydgoszcz, Nicolaus Copernicus University in Toruń, 85-092 Bydgoszcz, Poland

**Keywords:** *Acanthamoeba* keratitis, oxidative stress, redox imbalance, host–parasite crosstalk, reactive oxygen species, antioxidant defense systems, corneal inflammation, environmental exposure, environmental adaptation

## Abstract

*Acanthamoeba* keratitis is a severe, sight-threatening corneal infection caused by free-living amoebae widely distributed in soil, natural waters, and domestic or recreational water systems. Following entry into the corneal microenvironment, disease develops within a redox conflict in which host-derived reactive oxygen species (ROS) and reactive nitrogen species (RNS) may contribute to parasite control while simultaneously promoting inflammation and tissue injury. *Acanthamoeba* responds through an adaptable defense network involving superoxide dismutases, catalase, thioredoxin- and glutathione-dependent systems, peroxiredoxins, and mitochondrial mechanisms. However, most mechanistic evidence derives from in vitro trophozoite studies, whereas the contribution of individual redox pathways to mature cyst survival and persistence in the infected cornea remains poorly defined. This review critically integrates evidence on host oxidative and nitrosative responses, parasite antioxidant adaptation, bidirectional redox crosstalk, and redox-modulating therapeutic strategies. Current data support neither indiscriminate suppression nor uncontrolled amplification of reactive species. Instead, future interventions should achieve spatially, temporally, and stage-specifically controlled redox modulation that weakens parasite defenses while preserving corneal integrity and essential innate immune functions. Progress will require causal validation of redox targets, separate assessment of trophozoites and mature cysts, long-term excystation and regrowth assays, evaluation across clinical isolates and genotypes, and parallel assessment of host-cell toxicity in clinically relevant corneal models.

## 1. Introduction

*Acanthamoeba* spp. are ubiquitous free-living amoebae found in soil, freshwater, seawater, air, recreational and domestic water systems, contact-lens accessories, and healthcare-associated settings [1]. Their broad environmental distribution creates multiple opportunities for human exposure. Their life cycle comprises a metabolically active trophozoite and a dormant cyst that is highly resistant to adverse environmental conditions [1]. Although these amoebae normally persist outside the human host, selected strains can cause opportunistic infections, of which *Acanthamoeba* keratitis (AK) is the most clinically important. Exposure may occur through contact with contaminated water, soil, or improperly handled contact lenses, particularly when the corneal epithelium is compromised. Following entry into the corneal microenvironment, the parasite is exposed to epithelial barriers, inflammatory mediators, immune-cell-derived oxidants, nutrient limitation, and anti-amoebic treatment, all of which impose substantial metabolic and redox stress [2,3,4].

*Acanthamoeba* keratitis is a severe, sight-threatening corneal infection that commonly presents with ocular pain, epithelial abnormalities, and stromal infiltrates and may progress to corneal perforation or require surgical treatment in advanced cases. Early disease is frequently misdiagnosed as bacterial, fungal, or viral keratitis, delaying the initiation of appropriate anti-amoebic therapy and worsening prognosis [5,6]. Treatment may continue for several months and is complicated by the two-stage life cycle of the parasite. Trophozoites are motile and proliferative, whereas cysts are dormant, structurally resistant, and capable of surviving therapies that are active against trophozoites, thereby contributing to persistent or recurrent disease [4,5]. Therapeutic evaluation must therefore distinguish trophocidal from cysticidal activity and confirm whether treated cysts retain the capacity for excystation and subsequent trophozoite proliferation [5]. These clinical and biological challenges provide the rationale for investigating how *Acanthamoeba* withstands oxidative and pharmacological stress and for identifying more selective redox-based therapeutic strategies.

Reactive oxygen species (ROS) and reactive nitrogen species (RNS) occupy a dual position in the pathogenesis of AK. Neutrophils and macrophages recruited to the infected cornea are capable of generating oxidants during the respiratory burst, and experimental studies support a contribution of neutrophil-derived, myeloperoxidase (MPO)-associated extracellular mechanisms to the killing of *Acanthamoeba* cysts. Neutrophil extracellular trap (NET) formation may additionally contribute to trophozoite containment. At the same time, excessive or insufficiently localized neutrophil activation can intensify stromal inflammation, proteolytic injury, and corneal tissue destruction. Recent experimental evidence showing that strong, non-targeted C-X-C motif chemokine ligand 1 (CXCL1)-mediated neutrophil recruitment reduced parasite burden but produced severe stromal damage illustrates this balance between antimicrobial activity and host-tissue injury. However, the relative contributions of NADPH oxidase-derived ROS, MPO-generated oxidants, RNS, NETs, and non-redox mechanisms to parasite clearance in the infected cornea remain incompletely defined [7,8,9].

*Acanthamoeba* is not a passive target of host-derived oxidative stress but can activate adaptive antioxidant responses that may promote survival under hostile conditions. Exposure of *A. castellanii* trophozoites to hydrogen peroxide increases the activities of superoxide dismutase (SOD), catalase (CAT), glutathione peroxidase (GPx), and glutathione reductase (GR) and induces transcriptional upregulation of major antioxidant genes. Additional studies have identified an extensive redox network involving thioredoxin reductases, thioredoxins, peroxiredoxins, glutaredoxin- and glutathione-dependent pathways, and mitochondrial mechanisms that modulate oxidant production. This plasticity may enable the parasite to withstand inflammatory and pharmacological redox stress while also creating molecular dependencies that could be exploited therapeutically. However, most available evidence derives from axenic trophozoite cultures, recombinant enzymes, or experimentally induced oxidative stress, and the essentiality of individual redox pathways in mature cysts and infected corneal tissue remains largely unresolved [10,11,12].

Available evidence on redox biology in AK remains fragmented across studies of corneal innate immunity, parasite antioxidant defenses, mitochondrial metabolism, and redox-modulating interventions. This review integrates current knowledge on the environmental-to-host transition of *Acanthamoeba*, host-derived ROS and RNS, parasite antioxidant adaptation, bidirectional host–parasite redox crosstalk, and the therapeutic potential of both antioxidant and controlled pro-oxidant strategies. Particular emphasis is placed on distinguishing association from causation, trophozoite responses from cyst responses, and direct amoebicidal activity from host-tissue protection. The therapeutic objective should therefore be neither indiscriminate suppression nor uncontrolled amplification of reactive species, but spatially, temporally, and stage-specifically controlled redox modulation that weakens parasite defenses while preserving corneal integrity and essential innate immune functions. Accordingly, AK can be viewed as an environmentally acquired disease in which a free-living amoeba undergoes a rapid ecological and redox transition from external reservoirs to the nutrient-limited, immune-active corneal microenvironment.

### Literature Search Strategy

To identify the relevant literature, PubMed/MEDLINE, Scopus, and Web of Science were searched from database inception through 9 July 2026. Google Scholar was used as a supplementary search tool. Search terms included combinations of “*Acanthamoeba*”, “*Acanthamoeba* keratitis”, “oxidative stress”, “reactive oxygen species”, “reactive nitrogen species”, “antioxidant defense”, “superoxide dismutase”, “catalase”, “thioredoxin”, “glutathione”, “neutrophil”, “macrophage”, “corneal injury”, “photodynamic therapy”, and “corneal cross-linking”. Additional publications were identified by screening the reference lists of relevant original articles and recent reviews. Study selection was based on relevance to the mechanistic scope of the review, with priority given to English-language primary studies providing stage-specific, redox-related, therapeutic, or corneal-tissue outcomes. Relevant review articles were used to provide broader context and to identify additional primary studies. Because this was a narrative, mechanistically focused review, study selection was not conducted according to a formal systematic-review protocol, and no meta-analysis was performed.

## 2. Environmental-to-Host Transition and Redox Biology of *Acanthamoeba*

### 2.1. Biphasic Life Cycle and Environmental-to-Host Transition

*Acanthamoeba* is an opportunistic protozoan that is ubiquitous in the natural environment. The life cycle of *Acanthamoeba* consists of two alternating morphological stages: the active trophozoite and the dormant, resilient cyst form. These stages typically range from approximately 10 to 40 µm in diameter, depending on the developmental stage and strain [5,13].

The trophozoite represents the metabolically active stage, characterized by motility, phagocytic capability, and mitotic replication under favorable conditions [1]. It possesses distinctive, dynamic cytoplasmic projections known as acanthopodia, which facilitate locomotion and adhesion to surfaces and host cells [14]. During this stage, the amoeba feeds on microorganisms and suspended organic particulate matter [1]. In a clinical context, trophozoites constitute the principal tissue-invasive stage and directly contribute to corneal tissue damage during *Acanthamoeba* keratitis [4,13].

**Figure 1:**
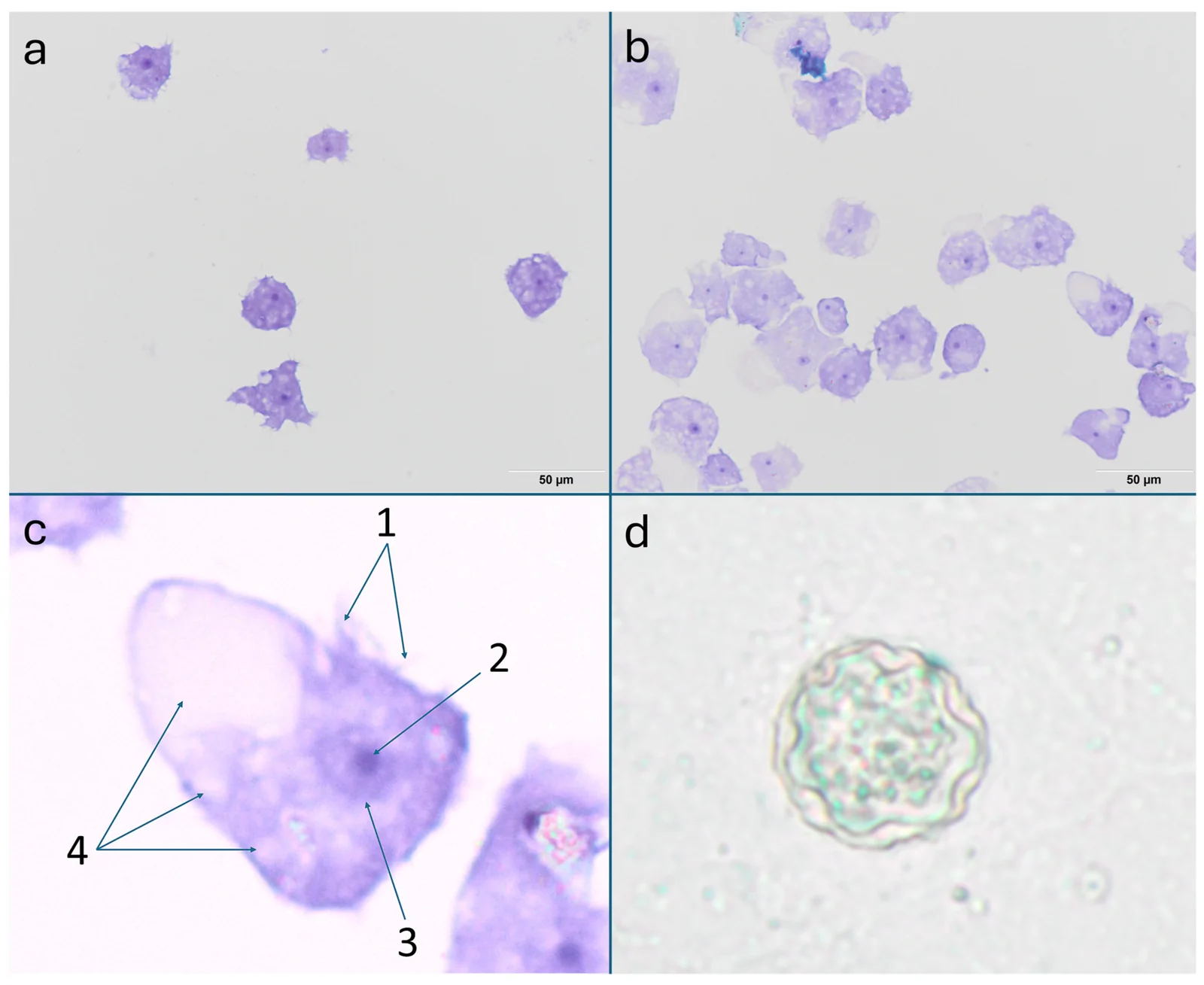
Representative morphological forms of *Acanthamoeba* from the authors’ laboratory material. (**a**) Isolated Giemsa-stained trophozoites displaying pleomorphic cell contours. (**b**) Field containing multiple Giemsa-stained trophozoites illustrating variability in cell size and morphology. (**c**) Enlarged view of a representative trophozoite highlighting characteristic structures: (1) acanthopodia, (2) nucleus, (3) nucleolus, and (4) cytoplasmic vacuoles. (**d**) Representative *Acanthamoeba* cyst observed in agar culture. Scale bars in panels (**a**,**b**): 50 µm.

Representative trophozoites and a cyst from the authors’ laboratory culture are shown in Figure 1 to illustrate the characteristic morphology of both developmental stages. The cells display variable, irregular contours, with fine acanthopodial projections visible along the cell periphery. A single round nucleus with a prominent nucleolus is discernible in many trophozoites, together with numerous cytoplasmic vacuoles.

The dynamic pleomorphism, cytoplasmic activity, and feeding behavior of a live *A. castellanii* trophozoite in the presence of *E. coli* are additionally shown in Supplementary Video S1.

The transformation of a metabolically active trophozoite into a metabolically dormant cyst form—termed encystment—is a complex differentiation process triggered by adverse microenvironmental conditions and accompanied by extensive transcriptional and structural remodeling. Oxidative stress may contribute to this response, but direct evidence that host-derived oxidants initiate encystment within the infected cornea remains limited [15]. During encystment, the amoeba activates stage-specific defense and structural programs, including the synthesis of cyst-wall components and cellulose synthase-dependent formation of the endocyst [16].

### 2.2. The Cyst Wall as a Physicochemical Barrier

When environmental conditions deteriorate, the amoeba transitions into the cyst stage, characterized by markedly reduced metabolic activity. This form enables *Acanthamoeba* to survive adverse environmental conditions, including desiccation, nutrient deprivation, and substantial fluctuations in temperature and pH. Cysts also show greater resistance than trophozoites to several physical and chemical stressors, including ultraviolet radiation [13,16].

A defining feature of the mature cyst is its double-layered wall, composed of an outer ectocyst and an inner endocyst [13,17]. The ectocyst contains protein and polysaccharide components, whereas the endocyst is a denser, cellulose-rich layer synthesized during encystment [16,17]. The two layers are separated by an intercystic space and are connected at specialized openings known as ostioles. The ectocyst is formed during the earlier stages of encystment, whereas formation of the endocyst and ostioles occurs later in the differentiation process [16,17]. Depending on the species and strain, mature cysts may exhibit polygonal, stellate, or more rounded morphology, reflecting the configuration of the inner endocyst and outer ectocyst [13,16,17].

This unique macromolecular architecture provides a physicochemical barrier that contributes substantially to the resistance of *Acanthamoeba* cysts to adverse environmental conditions, disinfectants, contact-lens sterilizing agents, and several anti-amoebic compounds [5,16,17]. It may also restrict the penetration or cellular uptake of some anti-amoebic compounds, although the permeability of the mature cyst wall to individual drugs and ROS has not been quantified systematically. Together with the markedly reduced metabolic activity of the cyst stage, this structure represents a major therapeutic obstacle and contributes to the persistence or recurrence of infection [5,13]. The biological plasticity of *Acanthamoeba*, combined with its capacity for reversible morphological transition in response to environmental conditions, further complicates effective treatment and complete parasite eradication [13,16].

### 2.3. Iron-Containing Superoxide Dismutase of Acanthamoeba castellanii (AcFe-SOD): Characterization and Functional Role

A major enzymatic component of the amoeba’s defense against radical-induced stress is an iron-containing superoxide dismutase of *Acanthamoeba castellanii* (AcFe-SOD), a member of the metalloenzyme family [18,19]. *Acanthamoeba castellanii* possesses two biochemically distinct superoxide dismutases: an iron-containing SOD and a copper–zinc SOD, rather than two AcFe-SOD isoforms [18]. AcFe-SOD catalyzes the dismutation of superoxide anions into hydrogen peroxide and molecular oxygen, thereby initiating subsequent steps of the antioxidant cascade. During infection, this activity may also contribute to the neutralization of superoxide generated by host immune effector cells [18,19].

AcFe-SOD is primarily localized within the cytosol of trophozoites [19]. Its expression and enzymatic activity increased under superoxide-generating conditions induced by paraquat or pyrogallol, whereas exposure to hydrogen peroxide did not significantly increase AcFe-SOD expression and partially inhibited its activity [19]. A later study reported increased total SOD activity and SOD transcript levels under H_2_O_2_-induced oxidative stress, although individual SOD isoforms were not distinguished [10]. Collectively, these findings support an important role for SOD-dependent defenses in the adaptation of *A. castellanii* to oxidative stress. However, the direct contribution of AcFe-SOD to resistance against neutrophil-mediated killing or to parasite survival within infected corneal tissue has not yet been demonstrated.

The presence of an iron-dependent superoxide dismutase in *Acanthamoeba* represents a potentially important biochemical distinction between the parasite and the mammalian host. Mammalian cells lack Fe-SOD and instead rely on evolutionarily distinct copper–zinc and manganese superoxide dismutases [18,19]. This divergence provides a rationale for considering AcFe-SOD as a potential target for selective low-molecular-weight inhibitors. In principle, inhibition of this enzyme could destabilize the parasite’s antioxidant shield and increase its susceptibility to oxidative stress. However, no selective AcFe-SOD inhibitor has yet been shown to combine anti-amoebic efficacy with adequate selectivity toward corneal epithelial cells or keratocytes.

Moreover, *Acanthamoeba* may be exposed not only to ROS but also to RNS. Activated macrophages can generate nitric oxide (NO), and experimental exposure to exogenous NO donors produced a dose-dependent reduction in the viability of cultured *A. castellanii* trophozoites [20]. Sodium nitroprusside exerted a stronger inhibitory effect than sodium nitrite, although it was also more cytotoxic to human corneal epithelial cells. NO-releasing silica nanoparticles likewise reduced amoebic viability without inducing visible encystment. NO donors also inhibited cyst-to-trophozoite transformation, suggesting possible activity against cysts, although a definitive cysticidal effect was not established. The precise intracellular mechanisms responsible for NO-mediated toxicity in *A. castellanii* remain unknown [20].

### 2.4. Alternative ROS Detoxification Systems and Thiol Mechanisms

The hydrogen peroxide generated within the trophozoite cytosol through AcFe-SOD activity remains potentially harmful to cellular structures, because in the presence of redox-active metal ions it can participate in Fenton chemistry and generate highly reactive hydroxyl radicals. To limit this threat, *Acanthamoeba* possesses additional enzymatic and thiol-dependent mechanisms for hydrogen peroxide detoxification, including catalase, peroxidases, and the thioredoxin and glutathione systems [10,11].

Catalase decomposes hydrogen peroxide into water and molecular oxygen and constitutes an important component of the antioxidant response of *A. castellanii*. Exposure of trophozoites to H_2_O_2_ was associated with increased CAT activity and increased CAT transcript levels [10]. Pharmacological inhibition of catalase-associated activity with 3-amino-1,2,4-triazole (3-AT) under oxidative-stress conditions was accompanied by increased parasite cell death, supporting a protective role for catalase. However, the high inhibitor concentration used and the absence of direct target-engagement measurements limit enzyme-specific interpretation of these findings.

Beyond catalase, *Acanthamoeba*’s defensive capacity is complemented by a thiol-dependent system involving peroxiredoxins (Prxs), thioredoxins (Trxs), and thioredoxin reductases (TrxRs). As non-heme peroxidases, Prxs use conserved cysteine residues to reduce hydrogen peroxide and other peroxide substrates. In a reconstituted enzymatic system, the small bacterial-type thioredoxin reductase AcTrxR-S used reduced nicotinamide adenine dinucleotide phosphate (NADPH) to reduce thioredoxin 1 (Trx-1), which subsequently reduced peroxiredoxin 1 (Prx-1) and methionine sulfoxide reductase A. Prx-1, in turn, catalyzed the reduction of hydrogen peroxide, thereby establishing a functional AcTrxR-S–Trx-1–Prx-1 pathway in *A. castellanii* [11]. By contrast, the physiological function of the large vertebrate-type thioredoxin reductase AcTrxR-L remained unresolved.

AcTrxR-S was strongly upregulated at both the transcript and protein levels following exposure to hydrogen peroxide or diamide, whereas AcTrxR-L was not significantly induced [11]. Subsequent transcriptional analyses demonstrated that the response of the thiol network depends on the nature of the oxidative challenge. Hydrogen peroxide strongly induced AcTrxR-S and Trx-1 but did not significantly increase the expression of the investigated Prxs or GPx, whereas diamide produced a broader transcriptional response involving both the thioredoxin and glutathione systems [21]. Consistent with the complexity of this network, RNA sequencing analysis of axenically cultured *A. castellanii* Neff trophozoites detected transcripts encoding 21 thioredoxins or thioredoxin-domain-containing proteins distributed across a broad range of expression levels [22]. However, the biochemical functions and contributions to oxidative-stress resistance of most of these proteins remain unresolved.

In parallel with the thioredoxin system, *Acanthamoeba* possesses a glutathione-dependent network involving reduced glutathione (GSH), GR, GPx, glutaredoxins, and glutathione S-transferases. Increased total GR and GPx activities were observed in trophozoites exposed to hydrogen peroxide [10]. Nevertheless, transcriptional activation of this system is not uniform: hydrogen peroxide had only limited effects on most of the investigated glutathione-system transcripts, whereas diamide markedly induced GPx and selected glutaredoxin- and glutathione-dependent components [21]. Auranofin exposure also stimulated several components of the glutathione system, suggesting that this pathway may partially compensate for disruption of thioredoxin-dependent redox homeostasis. Together, these findings indicate functional overlap and stressor-dependent coordination between the thioredoxin and glutathione networks rather than their uniform, simultaneous activation.

The maintenance of thiol-dependent antioxidant systems also requires an adequate supply of cysteine. *Acanthamoeba castellanii* expresses a functional cysteine synthase (AcCS), which catalyzes cysteine formation from O-acetylserine and sulfide. Expression of AcCS was downregulated following exposure to exogenous cysteine, consistent with feedback regulation of this biosynthetic pathway [23]. Because cysteine provides reactive thiol groups and serves as a precursor for glutathione and other sulfur-containing biomolecules, its biosynthesis may contribute to the maintenance of intracellular redox capacity. However, the direct contribution of AcCS to oxidative-stress resistance or survival within corneal tissue has not yet been experimentally established.

Redox regulation also appears to be involved in the transition from the trophozoite to the cyst stage. Transcriptomic profiling of experimentally induced encystment in an *A. castellanii* T4 strain demonstrated coordinated downregulation of genes encoding cytochrome-*c* oxidase, alternative oxidase, and alternative NADH dehydrogenases during the first 24–48 h, followed by their upregulation at 72 h. Several antioxidant-related enzyme families, including catalases, glutaredoxins, glutathione S-transferases, peroxiredoxins, and thioredoxins, also showed stage-dependent regulation, while the available glutathione pool decreased by approximately 40–60% during the first 24 h [15]. These findings support a model in which a transiently pro-oxidative intracellular environment contributes to the metabolic reorganization accompanying early encystment. However, intracellular ROS concentrations and the activities of most of the implicated enzymes were not directly measured.

## 3. Oxidative Stress in the Corneal Microenvironment During *Acanthamoeba* Keratitis

### 3.1. Host Inflammatory Response to Acanthamoeba Infection

The initiating phase of *Acanthamoeba* keratitis (AK) pathogenesis is the adhesion of the parasite to corneal epithelial cells (CECs). The parasite–host interaction begins with the protozoan’s recognition of specific glycoconjugates on the surface of CECs, which contain mannose-rich oligosaccharide residues. Their number increases on the surface of damaged CECs [24]. A key adhesion factor on the parasite side is the mannose-binding protein (MBP), located on the parasite surface, which binds host mannose residues and enables stable attachment to the corneal epithelium [3]. Engagement of MBP leads to the secretion of mannose-induced protease 133 (MIP-133) by *Acanthamoeba* trophozoites, which induces apoptosis of CECs and facilitates parasite penetration through Bowman’s membrane [4]. As the disease progresses, amoebae infiltrate the corneal stroma, triggering inflammatory reactions [2] that may lead to necrosis [25,26]. Another protease facilitating parasite invasion is plasminogen activator [27]. Both developmental stages contribute to AK in different ways. Motile trophozoites use acanthopodia for adhesion to host cells [14], and mediate active tissue damage, whereas non-motile, environmentally resistant cysts contribute to persistence and recurrent disease. Trophozoites are responsible for active tissue damage, whereas cysts are crucial for recurrent episodes of infection [8]. Various pathogenic microorganisms, including viruses, bacteria, and fungi, may also enhance the virulence of *Acanthamoeba,* as the amoeba serves as a vector and reservoir for these pathogens [28,29].

The course of the immune response in AK has not yet been fully elucidated. However, it has been demonstrated that innate immune mechanisms play the predominant role in controlling *Acanthamoeba* invasion. A key mechanism initiating and sustaining corneal inflammation is the activation of CECs. It has been shown that *Acanthamoeba* activates Toll-like receptors (TLRs) expressed on CECs, which recognize specific pathogen-associated molecular patterns (PAMPs), leading to the initiation of the inflammatory response in the cornea [30]. Experimental studies have demonstrated that stimulation of human and Chinese hamster CECs with *Acanthamoeba* trophozoites leads to increased expression of TLR4, confirming its involvement in the recognition of *Acanthamoeba* [30]. The role of TLR4 is to activate CECs, which in turn begin to secrete chemokines and pro-inflammatory cytokines responsible for recruiting neutrophils into the corneal stroma. An increase in interleukin-8 (IL-8) messenger RNA (mRNA) expression has also been observed in human corneal epithelial cells (HCECs) following exposure to *A. castellanii* [30]. IL-8 is a pro-inflammatory cytokine capable of activating neutrophils, leading to enhanced generation of ROS, which may consequently result in oxidative stress [31].

Under in vitro conditions, both trophozoites and cysts of *A. castellanii* have been shown to induce the expression of IL-1α, IL-6, IL-8, and CXCL1 in HCECs. Notably, elevated mRNA levels of IL-1α, IL-6, and IL-8 were observed as early as 3 h after contact between the cells and the parasite, highlighting the rapidity of this response [32]. The epithelial response initiates a chemotactic cascade that leads to the rapid influx of neutrophils and macrophages. These cells constitute the first line of defense against the parasite and are considered key components of the innate immune response [33]. Neutrophils and macrophages can generate ROS during the respiratory burst, and oxidant-dependent effector mechanisms have been proposed to contribute to anti-*Acanthamoeba* defense [34]. However, their relative contribution to parasite killing during AK remains incompletely defined. The role of macrophages likely involves the direct killing of trophozoites on the ocular surface [33]. Neutrophils are also capable of killing *Acanthamoeba* trophozoites, as demonstrated in in vitro studies [35]. It has additionally been confirmed that both macrophages and neutrophils can destroy *Acanthamoeba* cysts, although they do not exhibit chemotaxis toward intact cysts. In vitro, neutrophil-derived extracellular factors showed cysticidal activity consistent with an MPO-associated mechanism, whereas macrophage-mediated cyst killing was dependent on phagocytosis [7]. In vitro studies have shown that neutrophils can release NETs into the extracellular space upon interacting with *Acanthamoeba*, enabling parasite killing and limiting its spread [8]. NET formation by neutrophils has been demonstrated exclusively in response to trophozoites; cysts did not induce NET release [8]. NETs are generated when pathogens are too large to be phagocytosed by neutrophils [36]. Although neutrophils and macrophages are essential for effectively eliminating parasites following *Acanthamoeba* infection, they may also contribute to corneal damage through the release of proteases [2]. In various ocular diseases, neutrophils exhibit a dual role: they perform protective functions, yet their excessive activation and infiltration, along with the release of pro-inflammatory cytokines, ROS, and NETs, can exacerbate tissue injury [37]. Studies conducted in the context of AK also indicate stromal necrosis resulting from neutrophil activity [38].

The recruitment of neutrophils and macrophages is also supported by the pro-inflammatory cytokine IL-17A. Studies in mice have shown increased expression of this cytokine in AK, and it has been demonstrated that IL-17A plays an important role in protecting the host against the parasite [39].

The complement system also participates in host defense against *Acanthamoeba* [40]. The mechanism of its activation in response to parasitic invasion has not yet been fully elucidated. The complement system may be activated through the classical, lectin, or alternative pathway. In the studies by Pumindonming et al. [41], *Acanthamoeba* was observed to bind the C3 and C9 components, activating the complement system primarily via the alternative pathway. Complement activation and the attachment of the C3b fragment to the trophozoite surface promote the recruitment of phagocytic cells [42].

Recent studies indicate that *Acanthamoeba* may modulate the host inflammatory response, representing an example of immune-evasion mechanisms employed by this parasite. It has been shown, among others, that AK is characterized by heterogeneity in both the number and function of neutrophils infiltrating the cornea [9], which may influence the course of the inflammatory response and the extent of tissue damage. In patients with AK, compared with individuals without AK, reduced expression of chemokines involved in neutrophil chemotaxis, such as CXCL1, C-X-C motif chemokine ligand 8 (CXCL8), and tumor necrosis factor (TNF), has also been observed, which may limit the effectiveness of neutrophil recruitment to the site of parasitic invasion [9].

### 3.2. Generation of Reactive Oxygen Species During Acanthamoeba Infection

Phagocytic cells, such as neutrophils and macrophages, employ a broad array of tools to eliminate pathogens. One of these is the activation of the phagocyte nicotinamide adenine dinucleotide phosphate (NADPH) oxidase, which leads to the generation of ROS [43,44]. The phagocyte NADPH oxidase is the NADPH oxidase 2 (NOX2) complex, a member of the NADPH oxidase family of enzymes responsible for the respiratory burst, i.e., the process that generates ROS during phagocytosis [45]. This enzyme is composed of two transmembrane proteins—the catalytic NOX2 subunit (formerly gp91phox) and p22phox, which form a heterodimer historically referred to as flavocytochrome b_558_, functioning as the redox center of the phagocyte NADPH oxidase [46,47]. It also consists of cytosolic proteins: p47phox (phox: phagocyte oxidase), p40phox, and p67phox, as well as the small G-proteins Rac1 (in monocytes) or Rac2 (in neutrophils) [48]. Owing to the spatial separation of the NADPH oxidase components, the enzyme is dormant in resting cells [48]. Upon cell stimulation, flavocytochrome b_558_ becomes activated, and cytoplasmic components associate with the p22phox subunit, enabling full activation of the NADPH oxidase [49]. The active enzyme catalyzes the transfer of electrons from cytosolic NADPH to molecular oxygen, generating the superoxide anion (O_2_^•−^) [50] according to the reaction:

NADPH + 2O_2_ ↔ NADP^+^ + 2O_2_^•−^ + H^+^

The resulting O_2_^•−^ may subsequently undergo dismutation to hydrogen peroxide (H_2_O_2_). This process can occur spontaneously at the acidic pH of the phagosome or, in the cytosol, may be catalyzed by SOD [51]. In the Haber–Weiss reaction, in the presence of a transition metal (or in the Fenton reaction in the presence of iron), H_2_O_2_ can react with O_2_^•−^, generating the hydroxyl radical (OH^•^) [51]. In the presence of myeloperoxidase (primarily found in neutrophils), hydrogen peroxide is converted into hypochlorous acid (HOCl) in the presence of Cl^−^ ions [50]. The reaction between OCl^−^ and H_2_O_2_ can further generate singlet oxygen (^1^O_2_) [51]. The ROS produced by the NOX2 complex serve a defensive function, supporting the destruction of parasites by immune cells, but they also play an immunoregulatory role. Numerous studies confirm that excessive ROS generation can induce oxidative stress, leading to DNA damage, protein modifications, and lipid peroxidation, which may ultimately result in cellular injury [37]. On the other hand, it has also been shown that a complete absence of ROS disrupts the proper functioning of signaling pathways and may lead to impaired immune responses [52]. The development of NET structures by neutrophils likewise depends on ROS and NOX2 [43,52].

CECs may also contribute to local ROS production because they are the first host cells exposed to the parasite and activate inflammatory signaling pathways [32]. IL-6/STAT3 signaling can influence cellular redox balance and ROS production in other experimental systems [53,54]. However, direct evidence that this mechanism operates in CECs during AK is currently lacking.

### 3.3. Oxidative Damage to Corneal Cells: Evidence and Knowledge Gaps

Oxidative stress has been observed in many cases of human corneal disease [55]. The phenomenon manifests as a decrease in antioxidant capacity, an increase in lipid peroxidation, the induction of proinflammatory cytokines, and an increase in the activity of proteolytic enzymes (metalloproteinases, serine proteases) and oxidoreductases [55,56]. Oxidoreductases may contribute directly to ROS generation, whereas proteolytic enzymes may amplify oxidative and inflammatory tissue injury. The presence of reactive oxygen and nitrogen species, along with the action of proteolytic enzymes, has been shown to cause corneal degradation [56]. Examples include atopic keratoconjunctivitis [57], dry eye disease associated with Sjögren’s syndrome [58,59], and keratoconus [60]. An important feature is the heterogeneity of oxidative-stress marker profiles among human corneal diseases. For instance, in corneas afflicted with bullous keratopathy, elevated malondialdehyde (MDA) concentration was observed, accompanied by no substantial alterations in peroxynitrite (ONOO^−^) concentration. This phenomenon contrasts with observations in Fuchs’ dystrophy, where a decrease in MDA and an increase in ONOO^−^ levels were documented. Conversely, elevated MDA and ONOO^−^ concentrations have been documented in cases of keratoconus [61].

Oxidative stress is not solely associated with inflammation; it can also induce keratocyte apoptosis in the corneal stroma. This process may represent a partially nonspecific host response observed across corneal infections caused by viruses, bacteria, fungi, and *Acanthamoeba* [62]. Probably, keratocyte apoptosis and stromal-cell loss may be protective mechanisms that contribute to the recovery of corneal transparency [62].

The pathogenesis of AK may involve analogous processes [26], but direct evidence for oxidative stress in ocular acanthamoebiasis is lacking. According to the extant literature, oxidative stress has been observed in murine models of disseminated acanthamoebiasis. Łanocha-Arendarczyk et al. [63] examined 96 lung samples from mice with disseminated acanthamoebiasis, which were monitored for 24 days. Thirty infected mice were immunocompetent (A), and the same number were immunosuppressed pharmacologically with methylprednisolone (AS). The authors compared the results with the corresponding controls: healthy mice with normal immunity (*n* = 18, C) and healthy mice with immunity suppressed as in AS mice (*n* = 18, CS). The authors demonstrated increased MDA concentration in A mice on day 8 of infection, whereas on day 16 of acanthamoebiasis, MDA level was higher in both A and AS mice [63]. MDA serves as an indicator of oxidative stress because it forms via the free-radical oxidation of polyunsaturated fatty acids in lipids. It is a toxic compound that, when present in excessively high concentrations, can lead to cellular damage, resulting in inflammation, apoptosis, or cancer [64,65]. The authors of that study also noted reduced activity of antioxidant enzymes (SOD on day 8, GPx on days 8 and 24, and GR on day 24) in AS mice. However, they provided no information as to whether this reduction in enzyme activity correlated with MDA concentration or disease symptoms [63]. Comparable findings were observed in a similar experiment in the eyes of immunocompetent (↓ CAT on days 8 and 16, ↓ GR on day 16) and immunosuppressed (↓ GPx on day 8) mice (same study design: disseminated acanthamoebiasis, duration 24 days, A and AS + C and CS mice) [66]. However, the concentration of GSH increased in A mice on day 24 of infection and in AS mice on day 16 [66]. The pattern of antioxidant responses was not uniform: CAT activity in A mice was higher than in C and AS mice on day 16, whereas GPx activity was lower in A than in C mice on day 8 [63]. On the one hand, reduced antioxidant-enzyme activity may indicate impaired antioxidant capacity, enzyme consumption, or inhibition under conditions of sustained oxidative challenge. On the other hand, the partial increase in antioxidant capacity may reflect a compensatory response to disturbed redox homeostasis, although it does not independently establish excessive ROS production [65].

Taken together, the available findings support disruption of redox homeostasis in mice with disseminated acanthamoebiasis. Several findings are compatible with oxidative stress, although the available evidence remains indirect. Increased expression of cyclooxygenases 1 and 2 (COX-1, COX-2) has been observed in the lungs of immunocompetent mice with acanthamoebiasis [67]. COX activity and ROS share a reciprocal, reinforcing relationship, where oxidative stress induces COX expression, and COX activity produces ROS, creating an inflammatory loop. ROS, including hydrogen peroxide, have been shown to directly modulate the activity or expression of cyclooxygenases (COX-1 and COX-2), thereby impacting prostaglandin synthesis and promoting a cascade of events that can lead to vascular dysfunction, tissue injury, and chronic inflammation [68]; however, too high concentrations of reactive oxygen or nitrogen species may suppress COX activity [69]. Furthermore, in the kidneys of mice with acanthamoebiasis, elevated expression and concentration of NADPH oxidase 2 and 4 (NOX2, NOX4) proteins were observed. A similar outcome was observed in mice immunocompromised by intravenous administration of a corticosteroid (methylprednisolone) [70]. NADPH oxidases catalyze electron transfer from NADPH to molecular oxygen, generating superoxide, which can subsequently give rise to other ROS. Subsequently, this radical is further converted into other forms of ROS by other oxidoreductases [71]. In addition, immunocompetent mice exhibited augmented gene expression and elevated concentration of nuclear factor erythroid 2-related factor 2 (Nrf2) [70], a pivotal transcription factor that orchestrates the body’s cellular defense against oxidative stress, inflammation, and toxins [72]. Concurrently, there was an increase in the expression and production of the antiapoptotic protein B-cell lymphoma 2 (Bcl-2), along with decreases in the expression and production of proapoptotic proteins, including Bcl-2-associated X protein (Bax) and caspases 3 and 9 [70]. Conversely, in immunosuppressed mice, the outcomes were opposite, despite the increase in Nrf2 gene expression (↓ Nrf2 concentration, ↓ gene expression and concentration of Bcl-2, ↑ gene expression and concentrations of Bax, caspase 3, and caspase 9) [70]. Research has demonstrated that pathogenic *Acanthamoeba* spp. induce apoptosis in human corneal epithelial cells [73,74]. In light of the available evidence, host-cell apoptosis during *Acanthamoeba* invasion may involve oxidative stress and mitochondrial pathways; however, a direct causal relationship has not been demonstrated in infected corneal tissue. The aforementioned matrix metalloproteinases (MMPs), which are active during acanthamoebiasis [26], may also interact with redox and apoptotic pathways. Free radicals can activate MMPs through oxidation of regulatory cysteine residues [75,76], whereas activated MMPs may further promote ROS formation [77,78] and apoptotic signaling [79]. Whether a comparable feed-forward mechanism operates in AK remains unknown. ROS, in conjunction with MMPs, have been demonstrated to induce cell apoptosis by targeting apoptotic proteins, such as Bax, Bcl-2, and caspase 3 [80]. Chusattayanond et al. [81] demonstrated that *Acanthamoeba*, isolated from patients with keratitis, induced apoptosis in mouse neuroblastoma cells, which was dependent on caspase activation and facilitated by the overexpression of proapoptotic proteins in the mitochondrial pathway. However, this was a noncorneal cell model and does not establish the same mechanism in corneal epithelial cells or keratocytes. In a separate study, Tripathi et al. [82] elucidated the role of the cytosolic phospholipase A2α (cPLA2α) pathway in the apoptosis of Chinese hamster corneal epithelial cells, as observed in cases of AK, suggesting a contribution from cell membrane degradation. The same molecular pathway has been demonstrated to induce apoptosis of human corneal epithelial cells [74].

Local oxygen availability may represent an additional modifier of host–parasite interactions in AK. Because the cornea is avascular, its oxygen supply depends substantially on diffusion from the atmosphere and aqueous humor, while inflammation, edema, and tissue injury may further disturb the balance between oxygen supply and metabolic demand [83]. Importantly, the effect of hypoxia has been examined directly in the context of *Acanthamoeba*. In human corneal epithelial cells challenged with a clinical T4 isolate, experimental hypoxia (1% O_2_) significantly attenuated the production of IL-8 and IFN-β and reduced *Acanthamoeba*-induced TLR4 and MyD88 expression as well as NF-κB and ERK1/2 activation [84]. These findings indicate that reduced oxygen availability can modify the epithelial innate response to *Acanthamoeba* and may increase host-cell susceptibility to infection. However, they do not establish that comparable levels of hypoxia occur within the infected corneal stroma in vivo or determine how reduced oxygen availability affects parasite redox metabolism.

Severe AK may progress to extensive stromal destruction and, in rare cases, necrotizing scleritis or sclerokeratitis [85]. In such advanced inflammatory lesions, increased metabolic demand, edema, inflammatory-cell accumulation, and tissue disruption could plausibly generate spatial oxygen gradients. However, oxygen tension and molecular markers of hypoxia have not been measured in necrotizing AK lesions. Consequently, a relationship between local oxygen depletion, altered host redox responses, parasite persistence, and necrotizing progression remains hypothetical and requires direct experimental investigation.

At present, no experimental study has comprehensively characterized redox imbalance directly within the cornea during primary *Acanthamoeba* keratitis. In particular, there are no time-resolved in vivo data simultaneously assessing parasite burden, local ROS or RNS generation, lipid peroxidation, protein oxidation, glutathione redox status, and antioxidant-enzyme activity within defined corneal compartments. The relative contributions of epithelial cells, infiltrating neutrophils, macrophages, keratocytes, and the parasite itself to the overall redox environment also remain unknown.

This limitation is important because increased inflammatory-cell infiltration or altered antioxidant-enzyme activity does not by itself establish oxidative damage as a causal driver of corneal pathology. Oxidative changes may contribute to parasite killing, host-tissue injury, compensatory antioxidant adaptation, or all three processes simultaneously. Consequently, the proposed role of redox imbalance in AK remains biologically plausible and supported by indirect evidence, but it requires validation in models that combine spatially resolved redox measurements with stage-specific assessment of trophozoites and cysts. These uncertainties form the basis for the host–parasite redox model discussed in Section 4.

## 4. Host–Parasite Redox Crosstalk: A Proposed Pathogenetic Model

### 4.1. Host-Derived Oxidative Stress as an Anti-Acanthamoeba Defense Mechanism

Neutrophils constitute an important component of the protective innate response during *Acanthamoeba* keratitis. In a Chinese hamster model, neutralization of macrophage inflammatory protein 2 or systemic neutrophil depletion resulted in earlier onset, greater severity, and prolonged persistence of corneal disease. Conversely, enhanced neutrophil recruitment induced intense early inflammation but accelerated the subsequent resolution of keratitis [86]. Corneal myeloperoxidase activity changed in parallel with neutrophil recruitment; however, it was used primarily as a marker of neutrophil accumulation. Because NADPH oxidase activity, ROS generation, and myeloperoxidase-dependent killing were not manipulated directly, these experiments establish an overall protective role for neutrophils but do not identify oxidative burst as the responsible amoebicidal mechanism.

Stage-specific studies provide more direct evidence for a redox-related neutrophil effector mechanism. Intact *A. castellanii* cysts did not induce chemotaxis of neutrophils or macrophages, although both cell types migrated toward cyst lysates. Once contact was established, neutrophils eliminated substantially more cysts than macrophages, and conditioned supernatants from cyst-exposed neutrophils retained cysticidal activity. This activity was not reduced by inhibition of nitric oxide synthesis but was abolished by quercetin under conditions reported to inhibit myeloperoxidase, while the activity of exogenous human myeloperoxidase was inhibited in parallel [7]. These findings support an MPO-dependent component of extracellular neutrophil-mediated cyst killing. However, neither hydrogen peroxide nor hypochlorous acid was measured directly, quercetin is not fully selective for MPO, and cyst viability was not confirmed using long-term excystation assays. The study therefore does not establish a complete MPO–H_2_O_2_–halide mechanism in the infected cornea.

Recent human and experimental data further indicate that the magnitude and localization of neutrophil recruitment are critical determinants of outcome. Single-cell transcriptomic analysis of corneas obtained from patients with advanced AK revealed a markedly lower proportion of neutrophils and attenuated CXCL-associated signaling compared with bacterial and fungal keratitis [9]. In a mouse model, high-dose CXCL1 markedly reduced the amoebic burden but caused severe corneal damage, whereas an *Acanthamoeba*-targeted CXCL1–nanobody fusion protein produced more localized and sustained neutrophil recruitment, reduced the parasite burden, and improved corneal outcomes. These findings support deficient chemokine-mediated neutrophil recruitment as a potential contributor to persistent AK and demonstrate the importance of spatially controlled immune activation. However, the human analysis included only three late-stage AK cases, and the study did not determine whether neutrophil-mediated parasite clearance involved ROS, MPO, NETs, or other effector mechanisms.

Macrophages also contribute to the early control of corneal infection. Experimental activation of conjunctival macrophages markedly reduced the incidence, severity, and chronicity of keratitis, while activated macrophages killed substantially more trophozoites than resting cells in vitro [87]. Although macrophage activation was accompanied by increased nitric oxide production, inhibition of inducible nitric oxide synthase did not reduce trophozoite killing. In contrast, cytochalasin D almost completely abolished macrophage amoebicidal activity, and conditioned macrophage supernatants were not cytolytic. These findings indicate that macrophage-mediated killing is predominantly contact- and actin-dependent, consistent with a phagocytosis-associated mechanism, rather than being primarily mediated by secreted nitric oxide [87]. Macrophage activation by *A. castellanii* also involves parasite-driven inflammatory signaling. Trophozoites induce IL-12 and IL-6 production predominantly through TLR4–myeloid differentiation primary response 88 (MyD88)-dependent pathways, while *A. castellanii* cysteine protease 3 (AcCP3) promotes an M1-like phenotype, nuclear factor kappa B (NF-κB) activation, *Nos2* expression, and nitric oxide production in murine macrophages [88,89]. However, because pharmacological inhibition of inducible nitric oxide synthase did not reduce trophozoite killing in the earlier functional study, AcCP3-induced NO production should not be interpreted as evidence that NO is the dominant macrophage amoebicidal mechanism. Its contribution may instead involve inflammatory signaling or tissue injury, which remains to be tested in corneal models [87,88,89].

Neutrophil extracellular traps provide an additional mechanism of trophozoite containment. Human neutrophils released NETs after interaction with *A. castellanii* trophozoites, whereas cysts did not induce a comparable response. Neutrophil coculture reduced viable trophozoite recovery, and deoxyribonuclease treatment partially restored parasite survival, supporting a contribution of NET-associated structures to amoebicidal activity [8]. At the same time, trophozoites degraded NET-derived DNA and displayed greater ecto-nuclease and ecto-3′-nucleotidase activities than cysts. However, the causal involvement of the predicted 3′-nucleotidase/nuclease was not confirmed through gene silencing or selective inhibition, and the ROS dependence of *Acanthamoeba*-induced NET formation was not investigated.

Taken together, the available evidence supports stage-specific and highly context-dependent innate effector mechanisms. Neutrophil-derived MPO-associated activity may contribute to cyst killing, whereas macrophage-mediated elimination appears predominantly contact- and phagocytosis-dependent. However, direct evidence defining the relative contributions of NOX2-derived ROS, MPO-generated oxidants, RNS, NETs, and non-redox mechanisms in the infected cornea remains incomplete.

### 4.2. Adaptive Antioxidant Responses of Acanthamoeba

Oxidative stress is not merely a damaging factor for *Acanthamoeba* but also a stimulus triggering adaptive antioxidant responses. Experimental exposure of *Acanthamoeba castellanii* trophozoites of the T4 genotype to hydrogen peroxide resulted in significantly increased activities of SOD, CAT, GPx, and GR. This enzymatic response was accompanied by transcriptional upregulation of key antioxidant genes, with SOD and CAT expression increasing approximately 4.5-fold and 2.2-fold, respectively. These findings indicate that *Acanthamoeba* actively reinforces its antioxidant defense machinery in response to oxidative challenge rather than passively succumbing to ROS-mediated damage [10].

The antioxidant response of *A. castellanii* also appears to depend strongly on the biological context and the nature of the redox challenge. During intracellular infection with wild-type *Legionella pneumophila*, transient increases in the transcripts encoding GPx, GR, and AcTrxR-S were accompanied by decreased expression of glutaredoxin, AcTrxR-L, peroxiredoxin-2, and one Fe/Mn-SOD transcript. At a later stage of infection, amoebae exhibited reduced ROS-associated fluorescence and lower abundance of GSH, indicating selective remodeling rather than uniform activation of the redox network [90]. However, these changes occurred during a bacterium–amoeba interaction and may reflect manipulation of the amoebic host by the bacterial type IV secretion system; they cannot be directly extrapolated to parasite adaptation during keratitis.

In addition to classical antioxidant enzymes, mitochondrial energy-dissipating mechanisms may contribute to the control of oxidative stress in *A. castellanii*. *A. castellanii* uncoupling protein (AcUCP) was shown to mediate mild mitochondrial uncoupling when heterologously expressed in *Saccharomyces cerevisiae*. In a yeast strain lacking cytosolic SOD1, AcUCP expression reduced mitochondrial superoxide levels and substantially improved growth and survival during hydrogen peroxide-induced oxidative stress. These findings support a potential antioxidant role of AcUCP through limitation of respiratory-chain over-reduction and mitochondrial ROS formation. However, this function was demonstrated primarily in a heterologous yeast model and has not yet been confirmed by targeted disruption of AcUCP in *A. castellanii* [91].

Collectively, these findings indicate that antioxidant adaptation in *A. castellanii* is not a uniform response but a context-dependent network involving enzymatic defenses, thiol homeostasis, and mitochondrial control of ROS production. This plasticity may promote parasite survival under oxidative pressure while simultaneously creating vulnerabilities that could be exploited therapeutically.

### 4.3. Antioxidant Defense as a Potential Therapeutic Vulnerability

**Figure 2:**
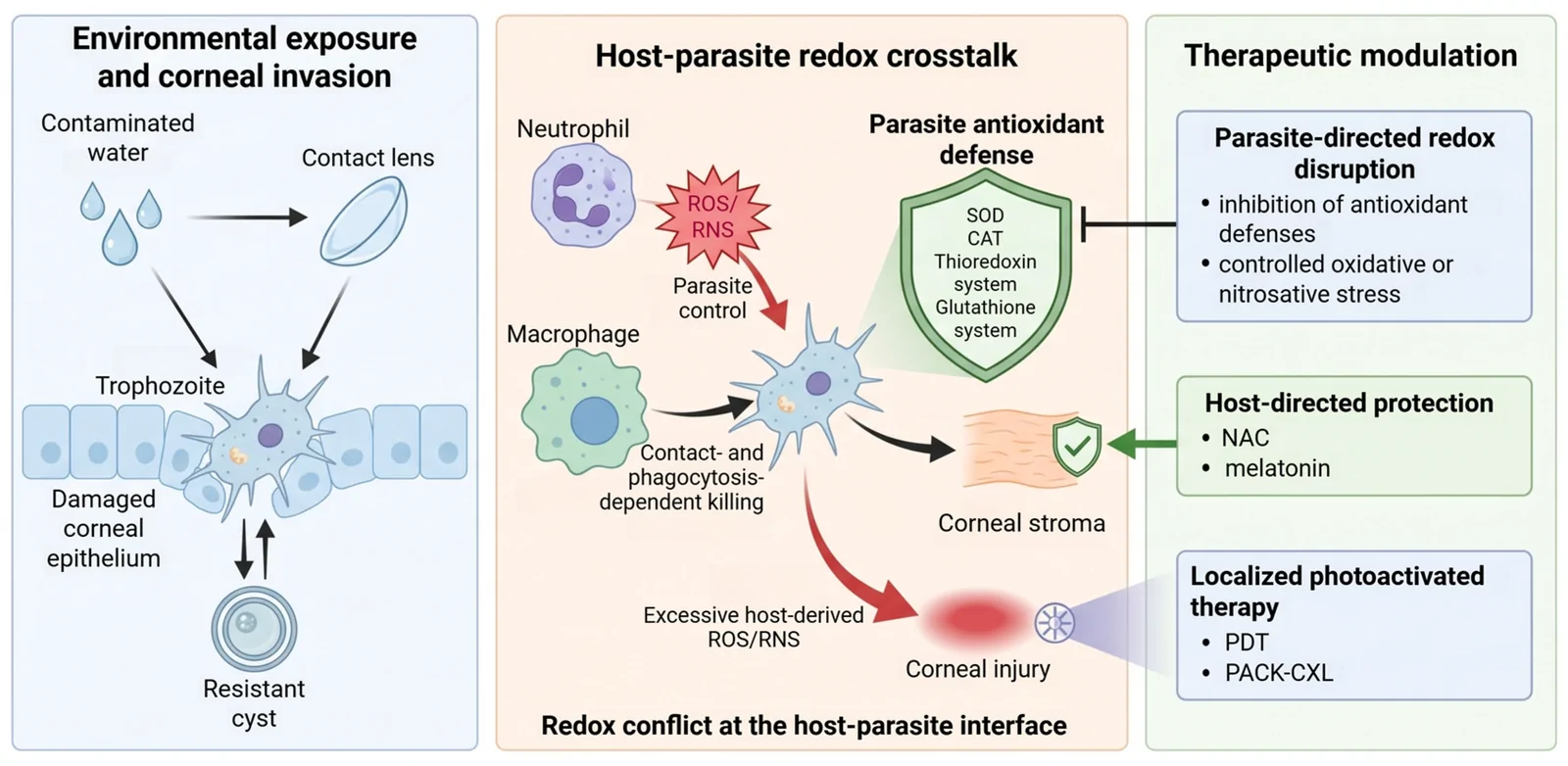
Proposed model of redox crosstalk in *Acanthamoeba* keratitis and its therapeutic modulation. Environmental exposure and disruption of the corneal epithelial barrier facilitate trophozoite adhesion and tissue invasion, whereas the cyst stage contributes to persistence and treatment resistance. Recruited neutrophils may generate reactive oxygen species (ROS) and reactive nitrogen species (RNS) involved in parasite control, while macrophage-mediated killing appears to be predominantly contact- and phagocytosis-dependent. Excessive or poorly localized oxidative responses may simultaneously aggravate stromal injury. *Acanthamoeba* counteracts redox stress through antioxidant defenses involving superoxide dismutase, catalase, and thioredoxin- and glutathione-dependent systems; however, current evidence derives predominantly from trophozoite studies, and the redox biology of mature cysts remains poorly defined. Potential therapeutic strategies include parasite-directed disruption of redox defenses, host-directed protection of corneal tissue, and localized photoactivated approaches. Photodynamic therapy primarily aims to achieve localized reactive-species-mediated parasite inactivation, whereas PACK-CXL may additionally stabilize the corneal stroma, although its direct amoebicidal activity remains uncertain. The figure presents a simplified conceptual model and does not imply equivalent levels of experimental evidence for all depicted mechanisms. Abbreviations: CAT, catalase; NAC, N-acetylcysteine; PACK-CXL, photoactivated chromophore for keratitis–corneal cross-linking; PDT, photodynamic therapy; RNS, reactive nitrogen species; ROS, reactive oxygen species; SOD, superoxide dismutase. Created in BioRender. Wesołowski, R. (2026) https://BioRender.com/i3uj4mt.

The dependence of *Acanthamoeba* on antioxidant enzymes may create a therapeutically exploitable vulnerability. Pharmacological attenuation of SOD and CAT activity during hydrogen peroxide exposure supported the involvement of these enzymes in oxidative-stress adaptation. However, marked Annexin V positivity and cell-cycle redistribution were observed primarily in the potassium cyanide (KCN)-treated group, and the limited selectivity of the inhibitors precludes target-specific attribution of these effects. These observations suggest that pharmacological interference with parasite antioxidant defense pathways may sensitize *Acanthamoeba* to host-derived or therapeutically generated oxidative damage. Moreover, parasite susceptibility may depend not only on the intensity of a single oxidative challenge but also on the cumulative burden of simultaneous oxidative and nitrosative stressors capable of overwhelming its protective systems [10,92]. However, these experiments provide mechanistic proof of concept rather than evidence of therapeutic efficacy: selective eradication of trophozoites or cysts has not been demonstrated in corneal models, and toxicity toward host corneal cells has not been assessed. An integrated overview of the environmental-to-host transition, host–parasite redox crosstalk, and the principal therapeutic modulation strategies is presented in Figure 2.

## 5. Therapeutic Implications

### 5.1. Current Therapeutic Approaches and Their Limitations

*Acanthamoeba* keratitis (AK) poses substantial therapeutic challenges because diagnosis is often delayed, the clinical course may be prolonged, and the cyst stage is markedly less susceptible than trophozoites to many available agents. Prompt initiation of intensive topical therapy is therefore essential. Common regimens begin with hourly administration, including overnight dosing during the first 48–72 h, followed by gradual reduction according to clinical response and ocular-surface toxicity. Treatment frequently continues for several months, although its duration should be individualized rather than defined by a fixed minimum period [6,13,93].

The therapeutic objective is to eliminate both trophozoites and viable cysts while limiting drug-induced corneal toxicity and controlling the host inflammatory response. In practice, these aims are difficult to reconcile because corneal penetration is variable, prolonged multidrug administration can damage the ocular surface, and reliable markers of microbiological eradication are lacking. Current management therefore combines anti-amoebic drugs, selected adjunctive agents, control of inflammation, and surgical intervention when medical therapy is insufficient.

#### 5.1.1. Biguanides and Diamidines

Biguanides remain the principal topical agents used in AK. Chlorhexidine and polyhexamethylene biguanide (PHMB), most commonly administered at 0.02%, are cationic compounds that interact with negatively charged membrane components, increase membrane permeability, and ultimately cause cell lysis. Both agents have activity against trophozoites and cysts, although susceptibility varies among isolates and mature cysts remain more difficult to eradicate. Diamidines, principally propamidine isethionate 0.1% and hexamidine 0.1%, also disrupt cellular membranes and interfere with macromolecular functions. Their activity against cysts is generally weaker and less predictable, and they are therefore commonly used in combination with a biguanide rather than as monotherapy [5,6].

Experimental ultrastructural studies provide a more detailed view of PHMB-induced injury in *Acanthamoeba*. In *A. castellanii*, high PHMB concentrations produced extensive trophozoite damage characterized by severe depletion of cytoplasmic contents, membrane-associated electron-dense precipitates, and a rough, granular cell surface. In cysts, PHMB caused swelling and partial withdrawal of the intracystic cytoplasm from the cyst wall [94]. Consistent findings were obtained in four corneal clinical isolates treated at minimum cysticidal concentrations of PHMB. Transmission electron microscopy demonstrated shrinkage of the intracystic amoeba, separation of its plasma membrane from the endocyst, cytoplasmic aggregation, deformation of intracellular organelles, reduction in mitochondrial cristae, nuclear-chromatin aggregation, and leakage of cytoplasmic material, whereas the ectocyst and endocyst remained comparatively well preserved [95]. These observations support the plasma membrane as a major site of PHMB-induced injury but also indicate substantial intracellular damage and relative structural persistence of the cyst wall.

Importantly, morphological or membrane damage should not automatically be equated with irreversible parasite killing. In *A. castellanii* strain 1BU, culture-based assessment after PHMB treatment demonstrated the appearance of smaller, irregularly shaped trophozoites after 72 h; these trophozoites subsequently failed to form new cysts during five weeks of observation. Survival estimates also differed substantially between enzymatic, dye-based, and culture-based viability assays, emphasizing the importance of post-treatment recovery and developmental competence when evaluating anti-*Acanthamoeba* activity [96]. Conversely, *A. castellanii* may engage survival mechanisms under PHMB pressure that are not dependent on encystation. In the Neff strain, CYP450 monooxygenase (CYP450MO) overexpression significantly increased survival following exposure to 0.01% PHMB at 1, 16, and 24 h, whereas expression of the encystation-associated genes *ATG8*, *CSI*, and *EMSP* did not differ significantly from control transfectants [97]. Although the authors proposed that CYP450MO may contribute to PHMB metabolism, direct biochemical biotransformation of PHMB by this enzyme was not demonstrated. CYP450MO should therefore be regarded as a candidate resistance-associated mechanism rather than a clinically validated PHMB-resistance determinant. Together, these observations highlight the distinction between acute structural damage, irreversible killing, and survival of a drug-exposed subpopulation. The distinction between in vitro susceptibility and treatment outcome is also clinically relevant: in the early PHMB series by Larkin et al. [98], cultured corneal isolates showed low PHMB cysticidal concentrations, yet one patient experienced treatment failure despite in vitro susceptibility, illustrating that laboratory susceptibility alone does not guarantee parasite eradication in the infected cornea.

In a prospective randomized comparison, chlorhexidine 0.02% and PHMB 0.02% monotherapy produced treatment success in 85.7% and 78.0% of evaluable eyes, respectively, with no statistically significant difference between the agents [99]. More recently, the phase 3 Orphan Drug for *Acanthamoeba* Keratitis (ODAK) trial showed that PHMB 0.08% monotherapy was non-inferior to PHMB 0.02% combined with propamidine 0.1%, with adjusted medical cure rates exceeding 86% in both groups when a standardized treatment protocol was followed [100]. These results provide the strongest current clinical evidence for PHMB-based therapy. Following the ODAK trial, polihexanide 0.08% was granted a European Union (EU)-wide marketing authorization as Akantior on 22 August 2024 for the treatment of *Acanthamoeba* keratitis in adults and children aged 12 years and older. At the time of its authorization, no medicine had previously been approved specifically for the treatment of AK in the European Union [101]. Nevertheless, intensive and prolonged exposure to biguanides and diamidines can cause ocular-surface toxicity, most commonly punctate epitheliopathy, and may complicate distinction between persistent infection and treatment-related epithelial damage. More severe adverse effects, including secondary elevation of intraocular pressure, have also been reported and may reflect cytotoxic injury to the trabecular meshwork [102].

#### 5.1.2. Antifungal Drugs

Azole antifungals have been investigated as alternative or adjunctive agents because inhibition of sterol 14-alpha-demethylase disrupts sterol biosynthesis and membrane function in *Acanthamoeba*. In a small randomized masked pilot trial, topical voriconazole 1% monotherapy produced reductions in infiltrate size comparable to those observed with PHMB 0.02% plus chlorhexidine 0.02%; however, only 18 patients completed the minimum treatment period, advanced cases were excluded, and the study was not powered to establish formal non-inferiority [103]. A retrospective series in which voriconazole 1% was added to PHMB and propamidine also reported favorable outcomes, but the contribution of voriconazole could not be separated from that of the concomitant first-line agents [104]. Voriconazole should therefore be regarded as a potentially useful adjunct, particularly in refractory disease, rather than a proven replacement for biguanide-based first-line therapy. Evidence for itraconazole, fluconazole, amphotericin B, and natamycin is more limited and is derived largely from in vitro studies or small clinical series [105].

#### 5.1.3. Corticosteroid Therapy

The use of topical corticosteroids in AK remains controversial. Although corticosteroids may reduce pain, inflammation, and immune-mediated corneal damage, their use before the diagnosis of AK or before the initiation of effective anti-amoebic therapy may worsen the clinical course. Experimental studies showed that dexamethasone enhanced excystation and trophozoite proliferation, increased parasite-mediated cytopathic effects on corneal epithelial cells, and aggravated the severity and chronicity of keratitis in an animal model [106].

Clinical evidence indicates that the timing of corticosteroid administration is critical. Corticosteroid use before the start of anti-amoebic therapy has been associated with poorer outcomes, whereas corticosteroids initiated after anti-amoebic treatment was established were not independently associated with an increased risk of treatment failure [107]. Accordingly, corticosteroids should not be used in suspected or untreated AK and, when required, should be introduced cautiously only in conjunction with effective anti-amoebic therapy. They may be considered in selected patients with persistent severe inflammation, scleritis, marked pain, or other inflammatory complications. However, the optimal corticosteroid agent, dose, timing, and duration of treatment remain undefined [102,107].

#### 5.1.4. Miltefosine and Salvage Pharmacotherapy

Oral miltefosine has emerged as a salvage adjunct for medically refractory AK. In a retrospective multicenter series of 15 treatment-resistant cases, all patients were considered free of active disease at the final follow-up, although miltefosine was administered together with other anti-amoebic agents and six patients required more than one course. Eleven patients developed increased ocular inflammation after treatment initiation, which improved with corticosteroids in most cases, while gastrointestinal symptoms were the most common systemic adverse effects [108]. These findings support a role for miltefosine in selected refractory cases, but controlled evidence is lacking and it should not be regarded as established first-line therapy.

#### 5.1.5. Antibiotics

Antibacterial agents are sometimes included in multidrug regimens, particularly when bacterial coinfection cannot be excluded or when reduction in the bacterial population serving as a nutritional source for trophozoites is considered desirable. Neomycin has historically been combined with propamidine, whereas polymyxin B, gramicidin, and other antibiotics have also been used in selected series [93,109]. However, their direct anti-*Acanthamoeba* activity is inconsistent and strain-dependent, and antibiotics should generally be regarded as adjunctive agents rather than reliable amoebicidal or cysticidal drugs [6,105].

#### 5.1.6. Surgical Treatment

Surgical intervention may be required when intensive medical treatment fails or when AK is complicated by progressive stromal destruction, impending perforation, or full-thickness corneal involvement [6,13]. Therapeutic epithelial debridement can remove superficially infected tissue, provide diagnostic material, and improve penetration of topical anti-amoebic agents, but it should be regarded as an adjunct rather than a reliably curative procedure [5,6]. Superficial ablative procedures, including phototherapeutic keratectomy, have also been described in selected cases; however, the supporting evidence remains limited, and complete eradication of cysts cannot be assumed [102,110].

When corneal transplantation is necessary, deep anterior lamellar keratoplasty (DALK) may be considered if the infection does not involve Descemet’s membrane or the endothelium, whereas penetrating keratoplasty (PKP) remains necessary in eyes with perforation, posterior corneal involvement, or full-thickness disease [6,13]. In a retrospective comparison, big-bubble DALK was associated with better three-year graft survival, preservation of endothelial-cell density, and visual outcomes than PKP, although patients undergoing PKP had more advanced disease at baseline [111]. Recurrence occurred after both procedures, emphasizing the importance of complete removal of infected tissue, continued perioperative anti-amoebic therapy, and cautious introduction of postoperative corticosteroids [111]. Optical keratoplasty may subsequently be required for visual rehabilitation after the active infection has been controlled [6].

Light-based adjunctive approaches, including photodynamic therapy and photoactivated chromophore for keratitis–corneal cross-linking (PACK-CXL), are discussed separately in Section 5.3.2 because their proposed effects are directly linked to the controlled generation of reactive oxygen species. Overall, current AK treatment remains prolonged, intensive, and potentially toxic, and no therapeutic regimen reliably eliminates mature cysts in every patient [5,6]. Variable drug penetration, incomplete stage-specific activity, inflammatory complications, and the frequent need for multimodal or surgical treatment provide a strong rationale for developing strategies that target parasite redox homeostasis or modulate redox-dependent host injury.

### 5.2. Targeting Parasite Redox Homeostasis

Experimental parasite-directed redox interventions, host-directed antioxidant approaches, and localized photoactivated strategies are discussed in Section 5.2 and Section 5.3. A detailed comparative synthesis of the experimental models, parasite stages, treatment protocols, anti-*Acanthamoeba* outcomes, host-cell or tissue effects, and major methodological limitations is provided in Table 1 at the end of Section 5.

#### 5.2.1. Disruption of Parasite Antioxidant Defenses

Among the antioxidant enzymes proposed as therapeutic targets in *Acanthamoeba castellanii*, iron-containing SOD is of particular interest because mammalian cells lack a canonical Fe-SOD counterpart. *A. castellanii* expresses both Fe-SOD and Cu,Zn-SOD [18], whereas subsequent functional studies demonstrated that the activity and expression of cytosolic AcFe-SOD increase under superoxide-generating conditions, supporting its role in trophozoite adaptation to oxidative stress [19]. However, basal antioxidant-enzyme activity should not be interpreted as a direct surrogate of pathogenicity. Comparative studies reported lower total SOD activity in some pathogenic or virulent strains than in avirulent or non-pathogenic isolates and found no consistent relationship between basal catalase activity and experimentally determined virulence [112,113].

Importantly, antioxidant enzymes considered as potential therapeutic targets are not uniformly parasite-specific. While *A. castellanii* possesses both Fe-SOD and Cu,Zn-SOD [18], mammalian ocular tissues lack Fe-SOD but contain CuZn-SOD, Mn-SOD, and extracellular SOD isoforms [114]. Thus, the absence of a mammalian Fe-SOD counterpart provides a biochemical rationale for considering AcFe-SOD as a potentially more divergent target, although this difference alone does not establish pharmacological selectivity. By contrast, several antioxidant systems present in *A. castellanii*, including CuZn-SOD, catalase, and thioredoxin- and glutathione-dependent pathways, have functional counterparts in host cells. In human corneal epithelial cells, CuZn-SOD, Mn-SOD, and catalase have been demonstrated experimentally [115], while TrxR, Trx, GR, and GPx activities have also been assessed in this directly relevant host-cell model [116]. Non-selective inhibition of these shared antioxidant systems could therefore impair host redox protection and narrow the therapeutic window. Candidate inhibitors should consequently be evaluated in parallel against parasite and human antioxidant enzymes and tested for toxicity toward relevant corneal cell types.

More recent pharmacological experiments support the involvement of SOD- and CAT-associated activities in adaptation to hydrogen peroxide; however, the limited selectivity of KCN and the high concentration of 3-AT preclude target-specific attribution and do not demonstrate selective trophocidal or cysticidal efficacy [10]. No selective AcFe-SOD or catalase inhibitor has yet been shown to eradicate trophozoites or mature cysts in a corneal model, and the essentiality and host-cell selectivity of these targets remain unconfirmed.

Beyond SOD and CAT, *A. castellanii* possesses an unusual thioredoxin system that includes a low-molecular-weight bacterial-type thioredoxin reductase (AcTrxR-S) and a high-molecular-weight vertebrate-type selenoprotein (AcTrxR-L). Biochemical studies demonstrated a functional AcTrxR-S–Trx-1 pathway linked to peroxiredoxin and methionine sulfoxide reductase activity, while AcTrxR-S was strongly induced by hydrogen peroxide and diamide, supporting its role as an adaptive component of the oxidative-stress response [11]. AcTrxR-L is also catalytically active and can reduce multiple thioredoxin-dependent substrates. In reconstituted systems, AcTrxR-L together with *A. castellanii* thioredoxin 1 (AcTrx1) additionally supported reduction of oxidized glutathione, suggesting functional overlap between the thioredoxin and glutathione networks, although the physiological relevance of this cross-system activity in intact amoebae remains unresolved [117].

Both thioredoxin reductases are pharmacologically inhibitable, but current evidence does not establish either enzyme as a validated intracellular drug target. AcTrxR-S was inhibited by auranofin and aurothioglucose in recombinant-enzyme assays, with IC_50_ values of 216 and 894 nM, respectively; however, whole-cell anti-*Acanthamoeba* activity was not assessed in that study [11]. Subsequent whole-cell studies demonstrated that auranofin inhibits the growth and viability of intact *A. castellanii* trophozoites. After 72 h, IC_50_ values of 2.97 µM and 3.48 µM were reported for the environmental Neff strain and the clinical JEA19 isolate, respectively, both belonging to genotype T4 [118]. Auranofin exposure was also associated with transcriptional changes in components of the thioredoxin and glutathione systems [21]. Nevertheless, these whole-cell observations do not demonstrate intracellular engagement of AcTrxR-S as the mechanism responsible for trophozoite killing.

For AcTrxR-L, auranofin and TRi-1 strongly inhibited the recombinant enzyme, with IC_50_ values of 0.32 and 0.36 µM, respectively, whereas TRi-2 showed substantially weaker enzyme inhibition (IC_50_ > 10 µM) [117]. In contrast, after 72 h of treatment, the corresponding trophozoite IC_50_ values were 5.64 µM for auranofin, 4.29 µM for TRi-1, and 2.08 µM for TRi-2 [117]. Thus, TRi-2 was the most active compound against trophozoites despite being the weakest inhibitor of recombinant AcTrxR-L. This discrepancy indicates that whole-cell activity cannot be attributed solely to AcTrxR-L inhibition and may involve additional molecular targets, differences in cellular uptake, or intracellular metabolism.

Host-cell selectivity also differed markedly among the compounds. At 72 h, TD_50_ values in human corneal epithelial cells were 0.97 µM for auranofin, 8.40 µM for TRi-1, and 8.08 µM for TRi-2, whereas the corresponding trophozoite IC_50_ values were 5.64, 4.29, and 2.08 µM, respectively. The resulting therapeutic indices (TD_50_/IC_50_) were 0.17 for auranofin, 1.96 for TRi-1, and 3.88 for TRi-2 [117]. Thus, auranofin showed an unfavorable selectivity profile, with HCEC toxicity occurring at a lower concentration than that required for 50% inhibition of trophozoites, whereas TRi-1 and TRi-2 showed only limited in vitro selectivity. These findings further emphasize that biochemical inhibition of a parasite redox enzyme does not necessarily translate into parasite-selective whole-cell activity.

Whole-cell studies further indicate that anti-*Acanthamoeba* activity is not a general property of gold(I) compounds but appears to depend strongly on their chemical scaffold. Phosphanegold(I) thiolates showed no significant effects on trophozoite viability, proliferation, encystation, or excystation, whereas more recent adamantane–azole gold(I) complexes displayed marked anti-trophozoite activity and reported synergy with chlorhexidine in selected combinations [119,120]. However, involvement of the thioredoxin system was inferred rather than demonstrated through direct target-engagement studies in intact amoebae, and activity against mature cysts was not established. The latter complexes should therefore be regarded as promising gold(I)-based leads rather than validated parasite-selective thioredoxin reductase inhibitors.

Mitochondrial and proteomic studies further indicate that *A. castellanii* can respond to redox disruption through partially redundant and adaptable pathways. The parasite can redirect electron flow between cytochrome-*c* oxidase and alternative oxidase, while prolonged inhibition of either pathway arrests trophozoite growth without necessarily causing irreversible killing [121]. Proteomic analyses of trophozoites exposed to different oxidative and nitrosative stressors also revealed coordinated changes in glutathione metabolism, thioredoxin-dependent defense, peroxiredoxins, NADPH-dependent reductases, and other oxidoreductases [12]. Together, these findings suggest that inhibition of a single antioxidant or respiratory pathway may be partly bypassed by compensatory mechanisms. Combination strategies may therefore be required, although the essentiality of individual targets and their relevance in mature cysts and corneal models remain to be established.

#### 5.2.2. Induction of Parasite Redox Imbalance

An alternative to inhibiting individual antioxidant enzymes is to drive the parasite beyond its compensatory capacity by increasing intracellular oxidant burden or disrupting mitochondrial bioenergetics, thiol metabolism, and metal-ion homeostasis. Chlorhexidine exposure has been associated with redox and metabolic remodeling in *Acanthamoeba*. In *A. polyphaga* trophozoites, treatment increased cytosolic antiradical capacity and was associated with reduced SOD-related and mitochondrial NADH-fumarate reductase activities, whereas succinate dehydrogenase activity remained unchanged [122]. Because no direct viability endpoint, ROS measurement, purified-enzyme assay, cyst model, or corneal-cell assessment was included, these findings do not establish enzyme-specific inhibition or a causal redox mechanism of parasite killing.

Nitroxoline provides broader evidence that pharmacological stress can destabilize multiple components of *Acanthamoeba* redox and metabolic homeostasis. Rodríguez-Expósito et al. [123] reported low-micromolar activity against trophozoites and cyst preparations across six *Acanthamoeba* strains. In mechanistic assays, treatment was associated with increased ROS-sensitive fluorescence, mitochondrial depolarization, ATP depletion, membrane and cytoskeletal alterations, and features compatible with apoptosis- and autophagy-like cell death. However, activity against cyst preparations was assessed metabolically without washout, excystation, or regrowth confirmation.

In *A. castellanii* ATCC 30011, Chen et al. [124] directly assessed intracellular oxidant-associated changes using DCFH-DA flow cytometry and demonstrated a dose-dependent increase in ROS-sensitive fluorescence following 24 h of nitroxoline exposure. Treatment was also associated with mitochondrial membrane depolarization, increased apurinic/apyrimidinic DNA lesions, upregulation of DNA-damage-response and antioxidant genes, reduced intracellular Fe^2+^ and total iron levels, and decreased endogenous hydrogen sulfide production [124]. Nitrosative-stress markers were not assessed. Following exposure of mature cyst preparations to 40 µM nitroxoline for 72 h, no detectable excystation or trophozoite regrowth was observed during the subsequent nine-day drug-free recovery period [124]. These findings support multifactorial genotoxic, oxidative, mitochondrial, and metabolic stress rather than selective inhibition of a single molecular target. However, the observed increase in ROS-sensitive fluorescence remains associative: no ROS-scavenging, antioxidant-rescue, or other functional intervention was performed to demonstrate that ROS generation was required for nitroxoline-mediated trophozoite killing. The predicted molecular targets, corneal-cell selectivity, and clinically achievable ocular exposure likewise remain unverified.

Isoliquiritigenin and glabridin provide further evidence that chemically induced redox stress can accompany mitochondrial and metabolic dysfunction. In *A. castellanii* ATCC 30011 trophozoites, both compounds produced dose- and time-dependent reductions in ATP-dependent metabolic viability, increased intracellular and mitochondrial oxidant-associated fluorescence, lowered mitochondrial membrane potential, and reduced SOD-associated activity in cell lysates [125]. Isoliquiritigenin additionally lowered the oxidized-to-reduced nicotinamide adenine dinucleotide ratio (NAD^+^/NADH) and altered transcriptional pathways associated with NAD^+^ metabolism, whereas glabridin affected sterol-related gene expression and reduced 7-dehydrocholesterol levels. These findings are consistent with pleiotropic redox, mitochondrial, and metabolic injury but do not establish ROS-mediated causality or direct molecular targets. Isoliquiritigenin showed an apparent HCEC selectivity index greater than 2.9, whereas glabridin had a narrow margin of approximately 1.9. The study was limited to one laboratory strain and the trophozoite stage, without washout, regrowth, or mature-cyst assays.

Normobaric hyperoxia provides preliminary evidence that sustained exposure to high oxygen concentrations can impair *Acanthamoeba* trophozoite growth or metabolic activity. Continuous exposure of four T3 or T4 strains to a pure-oxygen atmosphere for 96 h produced a strain-dependent reduction in alamarBlue signal and qualitatively increased CellROX Deep Red fluorescence [126]. These findings are consistent with hyperoxia-associated oxidative stress but do not establish ROS-mediated causality or irreversible trophozoite killing, because oxidant-associated fluorescence was not quantified and no antioxidant rescue, oxygen dose–response, washout, or regrowth assays were performed. The prolonged exposure conditions, absence of cyst testing, and lack of corneal or in vivo validation substantially limit direct therapeutic translation.

#### 5.2.3. Redox Sensitization and Combination Strategies

The resistance of *Acanthamoeba* cysts to individual oxidants may be partly overcome by combining complementary redox-generating systems. In a contact-lens disinfection model, the addition of iodide and plant-derived peroxidases markedly enhanced hydrogen peroxide-mediated cyst killing, potentially through the formation of reactive halogen species such as hypoiodous acid [127]. Cyst viability was assessed through seven-day excystation and subsequent trophozoite replication, providing stronger evidence of cysticidal activity than membrane-integrity or metabolic assays alone.

Similarly, acidified sodium nitrite markedly enhanced the activity of hydrogen peroxide against *Acanthamoeba* trophozoites and cysts. In *A. polyphaga* strain Ros, a clinical isolate obtained from a case of AK in the United Kingdom, 0.4% H_2_O_2_ combined with 2 mg/mL acidified NaNO_2_ at pH 5 produced an approximately 4-log reduction in trophozoite viability within 15 min and a 4-log reduction in cyst viability within 1 h [128]. Cysticidal activity was additionally evaluated in six other *Acanthamoeba* strains obtained from keratitis cases, for which species and genotypes were not reported. In these strains, 0.4% H_2_O_2_ combined with 0.5 mg/mL acidified NaNO_2_ produced a 2–3-log reduction in cyst viability after 1 h and an approximately 4-log reduction after 2 h, with no reported variation in cyst susceptibility among the six strains [128]. Cyst viability was assessed after neutralization by excystation and subsequent trophozoite replication over up to 7 days. The effect was strongly pH-dependent, whereas peroxynitrite and other reactive nitrogen intermediates were proposed as possible mediators but were not measured directly.

These findings support the concept that simultaneous oxidative, nitrosative, or halogenative stress can exceed the protective capacity of the cyst stage. Nevertheless, both systems were developed for disinfection rather than treatment of established keratitis and relied on conditions that may be incompatible with corneal tissues. Their principal significance is therefore mechanistic: they demonstrate the potential of redox sensitization and combination strategies, while therapeutic translation will require physiological pH, parasite-selective delivery, and rigorous evaluation of corneal toxicity.

#### 5.2.4. Nitric Oxide-Based Approaches

Nitric oxide (NO)-donor systems provide proof of concept that nitrosative stress can impair *Acanthamoeba castellanii* viability. In a single-strain in vitro study, sodium nitrite and sodium nitroprusside produced concentration- and time-dependent reductions in trophozoite metabolic activity; sodium nitroprusside was more potent, leaving <10% viability after 7 days at 1 mM, but it also caused substantially greater toxicity to human corneal epithelial cells [20]. Branched-polyethyleneimine-coated NO-releasing silica nanoparticles were internalized into trophozoite vacuoles and reduced metabolic viability by >60% at 100 µg/mL, whereas corresponding NO-free nanoparticles showed no comparable anti-*Acanthamoeba* activity. All three delivery systems also reduced cyst-to-trophozoite transformation. However, continuous exposure, reliance on metabolic assays, absence of an NO-quenching control, and lack of post-washout excystation or long-term regrowth prevent interpretation of these findings as definitive trophocidal or cysticidal activity.

Combining nitric oxide donors with sodium hypochlorite further suggested that simultaneous nitrosative and oxidative stress can enhance in vitro anti-*Acanthamoeba* activity [92]. In *A. castellanii* ATCC 30010, 0.125 mg/mL sodium hypochlorite combined with 10 µM sodium nitrite reduced trophozoite metabolic viability by approximately 60% after 7 days without significantly reducing human corneal epithelial-cell viability and produced approximately 20% SYTOX-positive cysts. Sodium nitroprusside combinations produced greater anti-*Acanthamoeba* activity and >50% cyst SYTOX positivity at 10 µM, but were substantially more toxic to corneal epithelial cells and keratocytes. Because the experiments involved prolonged exposure and relied on metabolic and membrane-permeability endpoints without washout, excystation, or regrowth testing, neither combination established selective irreversible trophocidal or cysticidal activity.

### 5.3. Host-Directed and Local Redox-Modulating Strategies

#### 5.3.1. Antioxidant-Based Approaches

##### N-Acetylcysteine

N-acetylcysteine (NAC) is one of the most extensively investigated redox-modulating agents in ophthalmology. It acts both as a direct scavenger of reactive oxygen species, including hydroxyl radicals, hydrogen peroxide, and hypochlorous acid, and as a precursor of intracellular glutathione, thereby supporting endogenous antioxidant defenses. Through these complementary mechanisms, NAC may reduce oxidative damage to cellular lipids, proteins, and nucleic acids and contribute to the restoration of redox homeostasis in inflamed ocular tissues [129,130].

Beyond its antioxidant effects, NAC can modulate redox-sensitive inflammatory pathways, including nuclear factor kappa B (NF-κB) signaling and the associated production of pro-inflammatory cytokines. Topical NAC has also been investigated in experimental and clinical contexts involving corneal injury and ocular surface disease. Reported effects include inhibition of matrix metalloproteinases, attenuation of inflammation, preservation of epithelial integrity, and promotion of corneal epithelial repair [129].

However, direct evidence supporting the use of NAC in *Acanthamoeba* keratitis is currently lacking. Most available data originate from models of non-infectious corneal injury or other ocular surface disorders and therefore cannot be directly extrapolated to AK. Nevertheless, its combined antioxidant, anti-inflammatory, and tissue-protective properties make NAC a plausible candidate for adjunctive treatment aimed at limiting host-mediated oxidative injury. Future studies should determine whether such protection can be achieved without suppressing potentially protective oxidant-dependent innate immune mechanisms or interfering with pathogen clearance.

##### Melatonin

Direct evidence supporting melatonin treatment in *Acanthamoeba* keratitis is currently lacking. In non-infectious corneal models, however, melatonin protected epithelial cells and fibroblasts against oxidant-induced injury by reducing intracellular and mitochondrial ROS, preserving mitochondrial membrane potential and viability, limiting apoptosis, and increasing antioxidant defenses [131,132]. These effects have been associated with heme oxygenase 1 (HO-1) and nuclear factor erythroid 2-related factor 2 (Nrf2)-dependent signaling, restoration of autophagic flux, and improved mitochondrial quality control, although the relative contribution of individual pathways appears to depend on the experimental model [132,133,134].

Additional experimental studies suggest that melatonin may support corneal repair and limit secondary inflammatory injury. Melatonin-loaded polymeric micelles reduced oxidative damage and promoted PTEN-induced kinase 1 (PINK1)/Parkin-associated mitophagy in hyperosmolarity-exposed corneal epithelial cells and improved ocular-surface parameters in experimental dry eye [133]. Topical melatonin also accelerated corneal re-epithelialization through predominantly melatonin receptor 2 (MT2)-associated signaling in a rabbit wound model and reduced inflammatory, angiogenic, and epithelial-damage markers after experimental alkali injury [135,136]. These findings support a tissue-protective potential but derive exclusively from sterile injury or ocular-surface disease models and do not address parasite clearance.

Melatonin should therefore be regarded only as a candidate host-directed adjunct in AK rather than as an anti-*Acanthamoeba* agent. Oxidant-dependent neutrophil mechanisms, MPO-associated activity, and NET formation may contribute to parasite control, whereas broad suppression of oxidative and inflammatory responses could protect corneal tissue while favoring trophozoite or cyst persistence [7,8,86]. Future studies should determine whether melatonin or melatonin-based delivery systems preserve epithelial, stromal, and endothelial integrity without increasing parasite survival, impairing innate immune clearance, or antagonizing conventional anti-amoebic and pro-oxidant therapies. Treatment timing and local exposure are likely to be critical.

#### 5.3.2. Photoactivated and Cross-Linking Approaches

Photoactivated strategies use light in combination with a photosensitizer or chromophore to generate short-lived reactive species and, in the case of corneal cross-linking, to increase stromal resistance to enzymatic degradation. Their therapeutic value depends on achieving sufficient anti-amoebic activity or tissue stabilization without inducing excessive injury to corneal epithelial, stromal, or endothelial cells [137].

A recent systematic review of novel anti-free-living amoeba compounds identified photochemical inactivation as one of the most extensively investigated redox-based approaches against *Acanthamoeba*. Among thirteen photoactive compounds evaluated against trophozoites or cysts, seven showed promising trophocidal activity and one demonstrated promising cysticidal activity. However, the selectivity profiles of most candidates remain insufficiently characterized, and at least one highly potent photosensitizer also exhibited marked cytotoxicity toward non-target corneal cells. These findings emphasize that the therapeutic value of photochemical approaches depends not only on amoebicidal potency but also on a carefully defined treatment window that limits damage to host corneal tissues [138]. Representative experimental protocols, anti-*Acanthamoeba* outcomes, host-cell effects, and major study limitations are summarized in Table 1 at the end of Section 5.

##### Photodynamic Therapy (PDT)

Photodynamic therapy combines a photosensitizer with wavelength-matched light to generate short-lived reactive species capable of damaging microbial membranes, proteins, and nucleic acids. Experimental studies indicate that both trophozoites and cysts of *Acanthamoeba* can be affected, but efficacy varies substantially according to photosensitizer localization, light dose, parasite stage, and the method used to confirm viability. The available evidence therefore supports photodynamic inactivation as a heterogeneous platform rather than as a single, uniformly effective intervention.

Photodynamic studies with hypocrellin B and RLP068 provide proof of concept that both trophozoites and cysts can be affected, whereas eosin-based photosensitizers have so far shown only partial activity against trophozoites [139,140,141]. Hypocrellin B showed strong anti-amoebic activity but an unfavorable therapeutic window because corneal-cell phototoxicity occurred below the concentrations required for complete parasite inactivation [139]. RLP068 markedly inhibited short-term excystation, although irreversible cyst eradication and long-term regrowth were not established [140].

Methylene blue-mediated PDT showed marked in vitro activity against *A. castellanii* trophozoites across several isolates, but cysts were substantially less susceptible, with more than half remaining culture viable after the principal protocol [142]. At a lower methylene blue concentration, combined treatment enhanced the effects of PHMB and amphotericin B, but not voriconazole, although formal pharmacodynamic synergy was not established. A supplementary experiment reportedly showed no appreciable damage in mouse corneas with epithelial defects, but efficacy and safety were not evaluated in an infected corneal model [142]. A mannose-conjugated porphyrin reduced trophozoite numbers and blocked excystation during the 72-h observation period; however, mannose-dependent targeting and irreversible cyst killing were not demonstrated [143]. Its apparent protection of host cells was substantially greater when amoebae were pretreated than when amoebae and host cells were irradiated simultaneously, indicating that selective delivery remains unresolved.

In vivo evidence remains limited. In a rabbit model of AK, a single session of TONS504-mediated photodynamic antimicrobial chemotherapy produced a favorable clinical and histopathological response in 11 of 19 treated eyes (58%), and no amoebae were observed histologically in responder corneas; the remaining eight eyes showed persistent severe inflammation and stromal disorganization [144]. However, follow-up was limited to 6 days, the between-group difference in clinical scores was significant only on day 5, and post-treatment culture or quantitative parasite-burden results were not reported. The study therefore did not establish microbiological eradication, cyst clearance, or protection against recurrence. Overall, PDT remains a promising redox-based adjunct, but clinical translation will require a reproducible therapeutic window, separate confirmation of trophocidal and cysticidal activity, long-term regrowth testing, and simultaneous evaluation of epithelial, stromal, and endothelial toxicity.

##### PACK-CXL

Conventional riboflavin/UVA PACK-CXL has shown limited and inconsistent direct anti-*Acanthamoeba* activity. In a trophozoite-suspension assay, Mito et al. [142] found that riboflavin/UVA treatment at 10.8 J/cm^2^ left respiratory activity at 91.9% of untreated control, with no significant difference from the untreated, UVA-only, or riboflavin-only groups. Del Buey et al. [145] similarly found that mixed trophozoite–cyst cultures from one clinical and one environmental T4 isolate remained viable after riboflavin/UVA exposure at 5.4 or 10.8 J/cm^2^; the 60-min protocol produced only a modest reduction in migration and degradation of some cysts. Using 0.1% riboflavin in 20% dextran and 370-nm UVA irradiation at 5.4 J/cm^2^, Sharma et al. [146] likewise detected no growth-inhibition zone in an agar-based *Acanthamoeba* assay at 24 or 48 h. However, the isolate, inoculum size, and stage composition were not characterized quantitatively, and the assay measured macroscopic growth or migration rather than viable-cell reduction. Taken together, these studies do not support reliable trophocidal or cysticidal activity of conventional riboflavin/UVA treatment.

More recent studies suggest that partial effects may occur under selected experimental conditions. Park and Park [147] found that riboflavin alone dose-dependently increased DCFDA fluorescence, whereas UVA alone reduced trophozoite metabolic viability to approximately 36–50% of control. Under continued post-irradiation culture in riboflavin-containing medium, 0.2% riboflavin combined with UVA reduced metabolic viability to approximately 8–22% over days 1–7, while 0.1% riboflavin plus UVA produced SYTOX Green positivity in approximately 55–60% of experimentally induced cysts. However, prolonged riboflavin exposure did not reproduce corneal pharmacokinetics and prevented attribution of the delayed effect to a single PACK-CXL session. Cyst maturity was not documented, SYTOX Green positivity was not confirmed by excystation or regrowth assays, and transcriptomic analyses were restricted to surviving cells. In a mixed inoculum containing approximately 80% trophozoites and 20% cysts, Atalay et al. [148] found that riboflavin/UVA produced trypan-blue positivity comparable to the untreated control, whereas 0.1–0.2% rose bengal activated with 523-nm green light increased the proportion of stained cells to 63–66%. However, the developmental stages were not analyzed separately, and immediate trypan blue staining without chromophore-only or light-only controls, washout, excystation, or regrowth testing did not establish irreversible trophocidal or cysticidal activity.

In vivo findings are less encouraging. In a rabbit model of AK induced by intrastromal injection of a human corneal isolate containing approximately 40% trophozoites and 60% cysts, riboflavin/UVA CXL performed on day 3 was associated four days later with higher clinical severity scores (3.48 versus 2.60) and higher parasite counts (285.8 versus 217.8 particles/µL) than no treatment [149]. However, the species and genotype were not reported, the inoculum was larger than would normally be expected clinically, follow-up was short, and untreated controls did not undergo sham epithelial debridement. The study therefore argues against CXL monotherapy under these experimental conditions but does not establish that cross-linking itself caused disease worsening or exclude a possible adjunctive tissue-stabilizing role alongside standard anti-amoebic therapy.

Clinical observations suggest possible adjunctive benefits but do not demonstrate direct parasite killing. In a single culture-confirmed, treatment-refractory case, PACK-CXL was followed by complete pain resolution within 4 weeks and gradual reduction in the infiltrate over the subsequent 10 weeks; at 3 months, no clinical signs of active infection were present, although central scarring, neovascularization, and poor visual acuity persisted [150]. Continued PHMB and chlorhexidine, multiple preceding interventions, and the absence of post-treatment microbiological testing prevented attribution of the outcome specifically to PACK-CXL or confirmation of parasite eradication. In a retrospective, non-randomized study of medically unresponsive AK, 11 eyes treated with CXL followed by lamellar keratoplasty were compared with 16 eyes undergoing lamellar keratoplasty without prior CXL. Two weeks after CXL, infiltrate area and depth decreased by 18.7% and 24.9%, respectively, and the confocal density of structures interpreted as cysts declined. At 1 year, recurrence occurred in 0 of 11 CXL-LK eyes versus 5 of 16 LK eyes, anti-amoebic treatment was shorter, and graft survival was higher in the CXL-LK group [151]. However, the small retrospective design, non-random treatment allocation, concomitant intensive pharmacotherapy and surgery, and reliance on confocal and histopathological morphology rather than culture-confirmed excystation or regrowth prevent attribution of direct cysticidal activity to CXL.

Potential benefits of PACK-CXL may therefore involve stromal stabilization, increased resistance to proteolytic degradation, confinement of corneal infiltration, or improved penetration of concomitant drugs rather than direct amoebicidal activity. Ex vivo studies in uninfected porcine corneas demonstrated increased resistance to collagenase digestion after riboflavin/UVA and rose bengal/green-light cross-linking, but parasite survival was not assessed [152]. Overall, conventional riboflavin/UVA PACK-CXL should be regarded as a potentially useful adjunct in selected refractory cases rather than as a validated stand-alone trophocidal or cysticidal treatment. Future studies should standardize chromophore exposure and irradiation parameters, evaluate trophozoites and mature cysts separately, confirm viability through long-term regrowth and excystation assays, and assess corneal-cell and tissue toxicity.

### 5.4. Integrated Therapeutic Perspective

Parasite-directed redox disruption, host-directed antioxidant protection, and localized photoactivated therapies should be viewed as complementary rather than mutually exclusive strategies. Interventions targeting parasite redox homeostasis seek to overwhelm or disable its adaptive defenses, whereas NAC, melatonin, and related host-directed approaches are intended primarily to limit excessive corneal injury. Photoactivated and cross-linking approaches may provide localized parasite inactivation, tissue stabilization, or both. The therapeutic objective should therefore be neither broad suppression nor indiscriminate amplification of reactive species, but controlled modulation that preserves corneal integrity while maintaining or enhancing parasite clearance.

Experimental studies in non-infectious corneal models support the tissue-protective potential of antioxidant interventions. Topical dimethylthiourea reduced inflammatory infiltration and improved corneal transparency after alkali injury, while serpin family A member 3K (SERPINA3K) limited hydrogen peroxide-associated ROS accumulation and apoptosis in corneal epithelial models [153,154]. However, these findings cannot be directly extrapolated to AK because suppression of ROS may also weaken oxidant-dependent innate immune mechanisms or reduce the efficacy of pro-oxidant therapies.

Conversely, sublethal oxidative exposure may promote adaptation rather than parasite eradication. *A. castellanii* trophozoites surviving sublethal calcium hypochlorite or plasma-activated water exposure displayed increased antioxidant-enzyme activity, altered glutathione homeostasis, enhanced expression of virulence-associated genes, and greater cytotoxicity toward macrophage monolayers [155,156]. Although these models do not reproduce corneal infection, they indicate that inadequately dosed pro-oxidant treatment may select or induce a more stress-tolerant phenotype. Future strategies should therefore combine parasite-directed redox disruption with spatially and temporally controlled protection of surrounding corneal tissues.

**Table 1:** Redox-modulating and photoactivated therapeutic strategies investigated against *Acanthamoeba*.

Strategy and Study	Experimental Model and Parasite Stage	Intervention or Protocol	Main Anti-*Acanthamoeba* Outcome	Host-Cell, Tissue, or Clinical Outcome	Key Limitation or Interpretation
Antioxidant-enzyme inhibition—Motavallihaghi et al. [10]	*A. castellanii* T4 cultures, primarily trophozoites, exposed to H_2_O_2_-induced oxidative stress	KCN and 3-AT, used to attenuate SOD and catalase activity, respectively, during H_2_O_2_ exposure	KCN-mediated attenuation of SOD activity was associated with marked Annexin V positivity and G0/G1 accumulation; 3-AT reduced catalase activity without a comparable increase in Annexin V positivity	Corneal-cell toxicity was not evaluated	Mechanistic evidence of antioxidant-enzyme involvement rather than selective trophocidal or cysticidal efficacy; inhibitor specificity, high concentrations, and inconsistent parasite-stage reporting limit target-specific interpretation
AcTrxR-S inhibition—Leitsch et al. [11]	Recombinant AcTrxR-S from *A. castellanii* Neff and a reconstituted AcTrxR-S–Trx-1 enzymatic system; no intact parasite stage was tested	Auranofin and aurothioglucose in recombinant-enzyme inhibition assays	No whole-cell anti-*Acanthamoeba* activity was assessed. Auranofin and aurothioglucose inhibited recombinant AcTrxR-S with IC_50_ values of 216 and 894 nM, respectively	Corneal-cell toxicity and selectivity over human thioredoxin reductases were not evaluated	Demonstrates biochemical inhibition and potential druggability of AcTrxR-S, but not intracellular target engagement, target essentiality, trophocidal or cysticidal activity, or parasite-selective efficacy. Subsequent whole-cell activity of auranofin was demonstrated independently [118], but without confirmation of intracellular AcTrxR-S engagement.
Auranofin—whole-cell anti-*Acanthamoeba* activity—Loufouma Mbouaka et al. [118]	*A. castellanii* Neff (environmental isolate) and JEA19 (clinical AK isolate), both genotype T4; trophozoites	Auranofin at 1–30 µM; trophozoite growth and viability assessed over 24–72 h	Dose-dependent inhibition of trophozoite growth and viability; 72-h IC_50_ values were 2.97 µM for Neff and 3.48 µM for JEA19	Host-cell toxicity was not evaluated	Demonstrates whole-cell activity of auranofin against both a laboratory strain and a clinical isolate, but does not establish intracellular engagement of AcTrxR-S or identify the molecular mechanism responsible for trophozoite killing; cyst activity was not evaluated
AcTrxR-L inhibition—Loufouma-Mbouaka et al. [117]	Recombinant AcTrxR-L and *A. castellanii* Neff T4 trophozoites	Auranofin, TRi-1, and TRi-2	Recombinant AcTrxR-L IC_50_ values were 0.32 µM for auranofin, 0.36 µM for TRi-1, and >10 µM for TRi-2. After 72 h, trophozoite IC_50_ values were 5.64, 4.29, and 2.08 µM, respectively. Thus, TRi-2 showed the strongest whole-cell activity despite being the weakest AcTrxR-L inhibitor.	At 72 h, HCEC TD_50_ values were 0.97 µM for auranofin, 8.40 µM for TRi-1, and 8.08 µM for TRi-2, corresponding to therapeutic indices of 0.17, 1.96, and 3.88, respectively.	The discordance between enzyme inhibition and trophozoite activity indicates that whole-cell efficacy cannot be attributed specifically to AcTrxR-L inhibition. Auranofin showed an unfavorable HCEC selectivity profile, while TRi-1 and TRi-2 showed only limited in vitro selectivity. Intracellular target engagement, target essentiality, and activity against mature cysts were not established.
Adamantane–azole gold(I) complexes—Israel et al. [119]	*A. castellanii* ATCC 50492 (T4) trophozoites	Gold(I) complexes C2, C3, and C4 alone and in combination with chlorhexidine	Marked anti-trophozoite activity; C2 and C4 induced phosphatidylserine exposure and cell-cycle alterations, while C4 also caused mitochondrial depolarization. Selected combinations with chlorhexidine showed reported synergy	C2 showed high apparent selectivity toward SIRC rabbit corneal cells, whereas C3 and C4 had narrow selectivity margins; the compounds were classified as non-irritant in the HET-CAM assay	AcTrxR inhibition and ROS-mediated killing were not demonstrated directly; docking used *Plasmodium falciparum* TrxR as a surrogate target. Only one strain and trophozoites were evaluated, without washout, regrowth, cyst, human corneal-cell, or infected-corneal studies
Chlorhexidine-associated redox disruption—Sifaoui et al. [122]	*A. polyphaga* ATCC 30461 (T4) trophozoites and subcellular fractions	Chlorhexidine (10 µM) followed by antiradical and enzyme-activity assays	No viability endpoint was assessed; treatment increased cytosolic antiradical capacity and was associated with reduced SOD-related and NADH-FRD activities, whereas SDH activity was unchanged	Corneal-cell toxicity was not evaluated	Suggests redox and metabolic disruption, but ROS production, direct enzyme inhibition, and a causal relationship with parasite killing were not demonstrated; cysts and regrowth were not assessed
Nitroxoline—Rodríguez-Expósito et al. [123]	Six *Acanthamoeba* strains; trophozoites and cyst preparations. Mechanistic assays used *A. culbertsoni* trophozoites and proteomics used *A. castellanii* L-10	Nitroxoline exposure followed by metabolic-viability, fluorescence-based mechanistic, and proteomic analyses	Low-micromolar activity against trophozoites and cyst preparations; increased intracellular ROS, mitochondrial depolarization, ATP depletion, membrane and cytoskeletal alterations, and features compatible with apoptosis- and autophagy-like cell death	Corneal-cell toxicity and ocular exposure were not evaluated	Cyst activity was not confirmed by washout, excystation, or regrowth assays; ROS-associated and cell-death changes did not establish causality or a direct molecular target
Nitroxoline—Chen et al. [124]	*A. castellanii* ATCC 30011 trophozoites and mature cyst preparations	Nitroxoline exposure followed by viability, transcriptomic, DNA-damage, redox, and post-treatment cyst-recovery assays	Reduced trophozoite metabolic viability, increased ROS-sensitive fluorescence, mitochondrial depolarization, phosphatidylserine exposure, and increased AP-site DNA lesions with activation of stress-response pathways. No excystation or regrowth was detected after 40 µM treatment during 9 days of drug-free recovery	Corneal-cell toxicity and ocular exposure were not evaluated	Supports genotoxic and redox-associated stress and sustained activity against mature cysts, but nitrosative-stress markers were not assessed and ROS-mediated causality was not established; predicted molecular targets were not experimentally validated, and only one strain and no corneal model were evaluated.
Isoliquiritigenin and glabridin—Chen et al. [125]	*A. castellanii* ATCC 30011 trophozoites; single laboratory strain	ISL and GLA exposure followed by metabolic-viability, redox, mitochondrial, transcriptomic, and host-cell cytopathogenicity assays	Reduced trophozoite metabolic viability, increased intracellular and mitochondrial oxidant-associated signals, mitochondrial depolarization, reduced SOD-associated activity, and apoptosis-like changes. ISL disrupted NAD^+^ homeostasis, whereas GLA altered sterol-pathway expression and reduced 7-dehydrocholesterol	ISL showed an apparent HCEC selectivity index >2.98, whereas GLA had a narrow margin of approximately 1.88; pretreatment of trophozoites reduced subsequent host-cell damage	ROS-mediated causality and direct NAD^+^- or sterol-pathway targets were not established. Only one strain and trophozoites were tested, without washout, regrowth, mature-cyst, or corneal-tissue studies; possible treatment-associated encystment was not evaluated
Normobaric hyperoxia—Sifaoui et al. [126]	Four *Acanthamoeba* strains representing T3 and T4; trophozoites	Continuous normobaric exposure to pure oxygen for 96 h	Strain-dependent reduction in alamarBlue signal and qualitatively increased CellROX Deep Red fluorescence	Corneal-cell, tissue, and in vivo effects were not evaluated	Supports hyperoxia-associated oxidative and metabolic stress but not irreversible trophozoite killing or ROS-mediated causality; oxygen dose–response, quantitative ROS analysis, washout, regrowth, and cyst assays were not performed
Peroxidase–H_2_O_2_–iodide system—Hughes et al. [127]	Mature cysts from nine *Acanthamoeba* strains in a contact-lens disinfection model	H_2_O_2_ combined with potassium iodide and plant-derived peroxidase, with or without a platinum neutralization disk	Markedly accelerated cyst killing across all tested strains, confirmed by 7-day excystation and trophozoite-regrowth assessment; activity was retained during platinum-mediated H_2_O_2_ neutralization	Corneal compatibility was not evaluated; residual H_2_O_2_ fell below 0.5 ppm by 4 h in the platinum-disk system	Strong culture-based evidence of cysticidal activity for contact-lens disinfection, but not for treatment of established keratitis. Hypoiodous acid formation was inferred but not quantified, and ocular safety of the complete system was not established
Acidified nitrite–hydrogen peroxide system—Heaselgrave et al. [128]	*A. polyphaga* strain Ros, a UK clinical AK isolate (trophozoites and cysts); cyst testing was additionally performed with six other keratitis isolates of unreported species/genotypes	Acidified NaNO_2_ combined with H_2_O_2_ at pH 5 in time-kill and contact-lens disinfection assays	In strain Ros, 0.4% H_2_O_2_ plus 2 mg/mL acidified NaNO_2_ produced approximately 4-log trophozoite reduction within 15 min and 4-log cyst reduction within 1 h. In six additional keratitis isolates, 0.4% H_2_O_2_ plus 0.5 mg/mL NaNO_2_ produced 2–3-log cyst reduction after 1 h and approximately 4-log reduction after 2 h, with no reported inter-isolate variation.	Corneal-cell and tissue compatibility were not evaluated; the system was intended for contact-lens disinfection	Relatively strong culture-based evidence of cysticidal activity, but efficacy was greatest at acidic pH, the prepared mixture was unstable, and the proposed peroxynitrite-mediated mechanism was not directly demonstrated
Nitric oxide (NO) donors and NO-releasing silica nanoparticles—Yim et al. [20]	Axenic *A. castellanii* ATCC 30010 trophozoites and experimentally induced cysts assessed in a cyst-to-trophozoite transformation model	Continuous exposure of trophozoites to sodium nitrite or sodium nitroprusside at 0.1–1000 µM, or 100-nm branched-polyethyleneimine-coated NO-releasing silica nanoparticles at 6.25–100 µg/mL, for 1–7 days; cyst assays used 1 mM donors or 100 µg/mL nanoparticles for up to 10 days	Sodium nitroprusside was the most active soluble donor, reducing trophozoite metabolic viability to <10% of control after 7 days at 1 mM. At 100 µg/mL, NO-releasing nanoparticles reduced viability by >60% from day 1, whereas NO-free nanoparticles lacked comparable activity. All three NO-delivery systems reduced cyst-to-trophozoite transformation	Sodium nitrite did not reduce human corneal epithelial-cell viability at concentrations up to 1 mM over 72 h. Sodium nitroprusside caused dose- and time-dependent cytotoxicity, while branched-polyethyleneimine-coated nanoparticles were cytotoxic at 100 µg/mL	Single-strain in vitro study using continuous exposure for up to 10 days. Metabolic-viability reduction and impaired cyst transformation did not establish irreversible trophozoite or cyst killing; no NO-quenching control, post-washout regrowth, long-term excystation, or corneal-tissue model was included
Nitric oxide donors combined with sodium hypochlorite—Park and Park [92]	Axenic *A. castellanii* ATCC 30010 trophozoites and experimentally induced cysts	Continuous exposure to sodium hypochlorite at 0.125 mg/mL combined with sodium nitrite or sodium nitroprusside at 0.1–100 µM for up to 7 days; cysts were assessed after 1–5 days	Sodium hypochlorite plus 10 µM sodium nitrite reduced trophozoite metabolic viability by approximately 60% after 7 days and produced approximately 20% SYTOX-positive cysts. Sodium nitroprusside combinations were more active, with >50% cyst SYTOX positivity at 10 µM	Sodium nitrite combinations caused no significant loss of primary human corneal epithelial-cell viability under the selected conditions. Sodium nitroprusside combinations produced marked time- and concentration-dependent toxicity to epithelial cells and keratocytes	Single-strain in vitro study using prolonged continuous exposure. Metabolic-viability reduction and SYTOX positivity did not establish irreversible trophozoite or cyst killing; no washout, excystation, regrowth, corneal-tissue, or in vivo assessment was performed, and no regimen achieved complete selective eradication
Hypocrellin B-PDT—Chen et al. [139]	A single clinical *Acanthamoeba* isolate obtained from a corneal ulcer; axenically cultured trophozoites and 7-day cyst preparations. Species and genotype were not reported	Hypocrellin B incubation for 4 h in the dark followed by >470-nm irradiation at 50 mW/cm^2^ for 30 min (90 J/cm^2^)	Concentration-dependent photoinactivation. No trophozoite growth was detected during 14-day post-treatment culture after exposure to 1 µg/mL for trophozoites or 20 µg/mL for cysts	Primary rabbit corneal epithelial and stromal cells showed nearly complete loss of viability at hypocrellin B concentrations above 0.156 µg/mL under the same irradiation conditions	The hypocrellin B concentrations that prevented post-treatment growth from trophozoites and cyst preparations (1 and 20 µg/mL, respectively) substantially exceeded those causing severe phototoxicity to primary rabbit corneal cells, indicating an unfavorable therapeutic window. Only one untyped clinical isolate was tested, without a corneal-tissue or in vivo infection model.
RLP068-mediated PDT—Ferro et al. [140]	Axenic *A. palestinensis* CCAP 1547/1; 4-day experimentally induced cyst cultures containing morphologically heterogeneous mature and immature forms	RLP068 at 0.5–5 µM for 1 h in the dark, followed by 600–700-nm irradiation at 50 mW/cm^2^ for 5–20 min (15–60 J/cm^2^)	Concentration- and fluence-dependent inhibition of excystation. At 5 µM and 60 J/cm^2^, excystation after 5 days in fresh medium was reduced to approximately 5% of the control, with cyst-wall disruption and cytoplasmic condensation	Corneal-cell and tissue toxicity were not evaluated	Short-term post-treatment excystation was assessed, but irreversible cyst eradication was not established. The cyst population was heterogeneous and partly immature, the endpoint could reflect both excystation and subsequent trophozoite proliferation, and ROS or singlet-oxygen generation was not measured directly
Methylene blue-mediated photodynamic therapy (MB-PDT)—Mito et al. [142]	*A. castellanii* ATCC 50370, originally isolated from an AK case; trophozoites and ≥2-week experimentally induced cysts. *A. castellanii* ATCC 30868 and four clinical AK isolates were additionally evaluated as trophozoites	Methylene blue (MB) at 0.5 mM for 10 min in the dark, followed by washing and 650–670-nm irradiation at 6 mW/cm^2^ for 30 min (10.8 J/cm^2^); combination experiments used 0.1 mM MB with PHMB, amphotericin B, or voriconazole	At 0.5 mM methylene blue, trophozoite respiratory activity and culture-based survival were reduced to 11.6% and 8.5% of control, respectively; additional reference and clinical-isolate trophozoites retained 0–27% respiratory activity. Cyst culture-based survival remained 55.4%. At 0.1 mM methylene blue, combined treatment enhanced the effects of PHMB and amphotericin B, but not voriconazole	A separate supplementary experiment in C57BL/6 mouse corneas with epithelial defects reportedly showed no appreciable corneal damage; quantitative corneal-cell toxicity and safety in infected corneas were not evaluated	Detailed efficacy studies focused on one strain, while additional isolates were assessed only by trophozoite respiratory activity. More than half of the cysts remained culture viable after the principal protocol. Efficacy was not tested in an infected corneal model, formal pharmacodynamic synergy was not established, and reactive oxygen species generation or the proposed molecular targets were not measured directly
Eosin Y (Red 22) and Red 28-mediated PDT—Vázquez-Ortega et al. [141]	Axenic *A. castellanii* Neff (ATCC 30010) trophozoites	Eosin Y (Red 22) or Red 28 at 250 µM during 2 h exposure to a 70-W white-light source	Under light exposure, Red 22 and Red 28 inhibited trophozoite growth by approximately 65% and >50%, respectively, after 96 h; Red 28 also showed appreciable dark activity. Qualitative imaging indicated increased membrane permeability and chromatin condensation	Host-cell and corneal-tissue effects were not assessed	Single-strain, trophozoite-only study using one concentration; light irradiance, spectrum, and fluence were not reported, and metabolic growth inhibition plus qualitative imaging did not establish irreversible killing
Mannose-conjugated porphyrin-PDT—Aqeel et al. [143]	Clinical *A. castellanii* ATCC 50492 (T4); trophozoites and cysts induced for up to 14 days on non-nutrient agar	Mannose-conjugated porphyrin at 10 or 50 µM for 1 h, followed by three washes and 465–480-nm blue-light exposure for 1 h	At 50 µM, trophozoite counts after 24 h were reduced by approximately 70%, whereas mannose or unconjugated porphyrin alone was inactive. Excystation was blocked during the 72-h observation period	Amoeba pretreatment reduced cytotoxicity toward human brain microvascular endothelial cells from approximately 95% to 4.9%; simultaneous treatment of amoebae and host cells reduced cytotoxicity from approximately 97% to 58%	Single-isolate in vitro study; reduced trophozoite counts did not distinguish irreversible killing from growth inhibition, and 72-h excystation blockade did not establish cyst eradication. Light irradiance and fluence were not reported, and mannose-dependent target engagement was not directly demonstrated
TONS504-mediated photodynamic antimicrobial chemotherapy (PACT)—Pertiwi et al. [144]	Japanese white rabbit model of AK induced with *A. castellanii* ATCC 30868-contaminated soft contact lenses	Topical TONS504 at 1 mg/mL followed by 660-nm light-emitting diode irradiation at 0.055 W/cm^2^, delivering 60 J/cm^2^ over 23 min with rest intervals	Eleven of 19 treated eyes (58%) were classified as responders after one treatment session; no *Acanthamoeba* were observed histologically in responder corneas	Responding eyes showed improved clinical appearance, more regular stromal organization, and lower neutrophil and macrophage infiltration; eight of 19 eyes showed an unfavorable response	Forty-two percent of eyes did not respond; follow-up was limited to 6 days, between-group clinical scores differed significantly only on day 5, and quantitative parasite-burden or post-treatment culture results were not reported
Riboflavin/UVA cross-linking comparator—Mito et al. [142]	*A. castellanii* ATCC 50370; trophozoites in suspension	Incubation in 0.1% riboflavin with 20% dextran for 60 min in the dark, followed by washing and 365-nm UVA irradiation at 3 mW/cm^2^ for 60 min (10.8 J/cm^2^)	Trophozoite respiratory activity remained at 91.9% of untreated control, with no significant difference from untreated, UVA-only, or riboflavin-only groups	Host-cell and corneal-tissue effects were not assessed	Single-strain, trophozoite-only suspension assay that did not model corneal cross-linking; cysts, culture-based survival, and post-treatment regrowth were not evaluated
Riboflavin/UVA PACK-CXL—del Buey et al. [145]	One clinical corneal isolate and one surface-water isolate, both genotype T4; mixed trophozoite–cyst inocula	0.1% riboflavin applied for 10 min before 370-nm UVA irradiation at 3 mW/cm^2^ for 30 or 60 min (5.4 or 10.8 J/cm^2^), with riboflavin reapplied every 5 min	Both isolates remained viable, with trophozoite growth beyond the treatment zone within 24 h and spread across the agar by 72 h. The 60-min protocol modestly reduced migration and caused degradation of some cysts	Corneal-cell and tissue effects were not assessed	Mixed-stage agar assay did not quantify stage-specific viable-cell reduction or model infected corneal tissue; only two T4 isolates were evaluated.
Riboflavin/UVA PACK-CXL—Park and Park [147]	Axenic *A. castellanii* ATCC 30010 trophozoites and experimentally induced cysts	Trophozoites were cultured with 0.005–0.2% riboflavin, with or without 30-min UVA irradiation at 3 mW/cm^2^ (5.4 J/cm^2^), and assessed over 1–7 days while riboflavin remained in the medium; cysts were treated with 0.1% riboflavin with or without UVA and assessed after 3 days	UVA alone reduced trophozoite metabolic viability to approximately 36–50% of control, whereas 0.2% riboflavin plus UVA reduced it to approximately 8–22% over days 1–7. Riboflavin dose-dependently increased DCFDA fluorescence, and 0.1% riboflavin plus UVA produced approximately 55–60% SYTOX Green-positive cysts	Host-cell and corneal-tissue toxicity were not evaluated	Single-strain in vitro study; prolonged post-irradiation exposure to riboflavin did not reproduce corneal pharmacokinetics and confounded attribution of the delayed effect to UVA treatment. Metabolic viability and SYTOX staining did not establish irreversible trophozoite or cyst killing; cyst maturity, excystation, and regrowth were not assessed
Riboflavin/UVA and rose bengal/green-light PACK-CXL—Atalay et al. [148]	Clinical *A. castellanii* T4 isolate from a severe AK case; mixed inoculum containing approximately 80% trophozoites and 20% cysts	Riboflavin at 0.1% or 0.25% with 365-nm UVA, or rose bengal at 0.1% or 0.2% with 523-nm green light; both protocols used 9 mW/cm^2^ for 10 min (5.4 J/cm^2^)	Riboflavin/UVA produced 11–14.5% trypan-blue-positive cells, comparable to 12% in the untreated control. Rose bengal/green light increased trypan-blue positivity to 63–66%, with no significant difference between the two rose bengal concentrations	Host-cell and corneal-tissue effects were not assessed	Single-isolate, mixed-stage assay in which trophozoites and cysts were not analyzed separately. Viability was assessed only by immediate trypan blue staining, without washout, excystation, or regrowth testing; light-only and chromophore-only controls were not included
Riboflavin/UVA PACK-CXL—Sharma et al. [146]	Uncharacterized *Acanthamoeba* culture on non-nutrient agar with an *Escherichia coli* overlay; trophozoites and cysts were observed, but their proportions were not reported	0.1% riboflavin 5-phosphate in 20% dextran applied for 20 min, followed by 370-nm UVA irradiation at 3 mW/cm^2^ for 30 min (5.4 J/cm^2^); UVA-only and riboflavin-only groups were also tested	No growth-inhibition zone was detected at 24 or 48 h after riboflavin/UVA, UVA alone, or riboflavin alone	Host-cell and corneal-tissue effects were not assessed	Species or strain, inoculum size, and stage composition were not reported. The agar-disc assay measured macroscopic growth or migration rather than quantitative viable-cell reduction, and vehicle and light-exclusion controls were incomplete
Riboflavin/UVA CXL monotherapy—Berra et al. [149]	New Zealand White rabbit model of AK induced by intrastromal inoculation of a human corneal *Acanthamoeba* isolate; inoculum contained approximately 40% trophozoites and 60% cysts	On day 3 after inoculation, a 7-mm epithelial debridement was followed by 0.1% riboflavin in 20% dextran for 30 min and 370-nm UVA irradiation at 3 mW/cm^2^ for 30 min (5.4 J/cm^2^), with riboflavin reapplied every 5 min	Four days after treatment, CXL-treated corneas had significantly higher parasite counts than untreated controls (285.8 ± 9.03 vs. 217.8 ± 7.22 particles/µL; *p* = 0.001), and viable trophozoites were recovered in culture	Clinical severity scores were higher in treated than untreated eyes (3.48 ± 0.30 vs. 2.60 ± 0.26; *p* = 0.008); histology showed intense inflammatory infiltration and cystic structures in treated corneas	Species and genotype were not reported. The model used an artificially high mixed-stage intrastromal inoculum, follow-up was limited to 4 days after treatment, histology included only one cornea per group, and untreated controls did not undergo sham epithelial debridement. The study evaluated CXL monotherapy rather than adjunctive use with standard anti-amoebic treatment
Adjunctive PACK-CXL—Watson et al. [150]	Single culture-confirmed case of prolonged, treatment-refractory human AK complicated by poor adherence and medication intolerance	After epithelial debridement, 0.146% riboflavin in 20% dextran was applied for 30 min; hypotonic riboflavin was used to increase corneal thickness to 400 µm, followed by UVA irradiation at 3 mW/cm^2^ for 30 min (5.4 J/cm^2^). Topical PHMB and chlorhexidine were continued	No post-treatment culture or quantitative parasite assessment was reported; no clinical signs of active infection were present at the 3-month follow-up	Severe ocular pain resolved completely within 4 weeks, and the infiltrate decreased in size and density over the following 10 weeks; central scarring, neovascularization, and poor visual acuity persisted	Single uncontrolled case with extensive previous and concomitant therapy. Continued PHMB and chlorhexidine, preceding voriconazole, miltefosine, and epithelial debridement, short follow-up, and absence of post-treatment microbiological testing prevent attribution of the outcome specifically to PACK-CXL or confirmation of parasite eradication
CXL before lamellar keratoplasty—Lin et al. [151]	Retrospective, non-randomized study of 27 eyes with medically unresponsive moderate-to-severe AK: CXL followed by lamellar keratoplasty (*n* = 11) versus lamellar keratoplasty without prior CXL (*n* = 16)	After epithelial debridement over the infiltrate, 0.1% riboflavin was applied every 5 min for six applications, followed by 370-nm UVA irradiation at 3 mW/cm^2^ for 30 min (5.4 J/cm^2^). Intensive anti-amoebic therapy was continued for 2 weeks before lamellar keratoplasty	Two weeks after CXL, infiltrate area and depth decreased by 18.7% and 24.9%, respectively, and the confocal density of cyst-like structures declined. Excised tissue showed lower cyst density and smaller, wrinkled structures than tissue from the keratoplasty-only group	At 1 year, recurrence occurred in 0 of 11 CXL-LK eyes versus 5 of 16 LK eyes; graft survival was 90.9% versus 68.75%, and anti-amoebic treatment duration was shorter (62.2 versus 182.8 days). Endothelial-cell loss was also lower in the CXL-LK group	Small retrospective, non-randomized study with potential selection bias. All patients received intensive pharmacotherapy and keratoplasty, preventing isolation of the CXL effect. Confocal and histopathological changes did not establish cyst death, and culture-confirmed excystation or long-term regrowth was not assessed

Abbreviations: 3-AT, 3-amino-1,2,4-triazole; AcTrxR-L, large vertebrate-type thioredoxin reductase of *Acanthamoeba castellanii*; AcTrxR-S, small bacterial-type thioredoxin reductase of *A. castellanii*; AK, *Acanthamoeba* keratitis; AP, apurinic/apyrimidinic; ATCC, American Type Culture Collection; ATP, adenosine triphosphate; CCAP, Culture Collection of Algae and Protozoa; CXL, corneal cross-linking; DCFDA, 2′,7′-dichlorodihydrofluorescein diacetate; GLA, glabridin; HCEC, human corneal epithelial cell; HET-CAM, hen’s egg test on the chorioallantoic membrane; IC_50_, half-maximal inhibitory concentration; ISL, isoliquiritigenin; KCN, potassium cyanide; LK, lamellar keratoplasty; MB, methylene blue; NAD^+^, oxidized nicotinamide adenine dinucleotide; NADH-FRD, NADH-dependent fumarate reductase; NO, nitric oxide; PACT, photodynamic antimicrobial chemotherapy; PACK-CXL, photoactivated chromophore for keratitis–corneal cross-linking; PDT, photodynamic therapy; PHMB, polyhexamethylene biguanide; ROS, reactive oxygen species; SDH, succinate dehydrogenase; SIRC, Statens Seruminstitut rabbit corneal cell line; SOD, superoxide dismutase; Trx-1, thioredoxin 1; TRi-1 and TRi-2, thioredoxin reductase inhibitors 1 and 2; TrxR, thioredoxin reductase; UVA, ultraviolet A.

## 6. Future Research Directions

### 6.1. Advanced Ocular Drug-Delivery Systems

Advanced ocular drug-delivery systems may facilitate the translation of anti-amoebic and redox-modulating compounds into clinically applicable therapies for *Acanthamoeba* keratitis. Nanoemulsions and microemulsions may improve the aqueous dispersibility of poorly soluble compounds, prolong their residence time on the ocular surface, enhance corneal penetration, and enable controlled drug release. These properties are particularly relevant to *Acanthamoeba* keratitis, in which prolonged treatment and frequent topical administration are often required. In a proof-of-concept study, a nanoemulsion containing a coumarin-rich extract from *Pterocaulon balansae* produced a concentration- and time-dependent reduction in the viability of *Acanthamoeba castellanii* T4 trophozoites. At a coumarin concentration of 1.25 mg/mL, approximately 95–98% loss of trophozoite viability was observed after 24 h, with an effect comparable to that of 0.1% chlorhexidine. However, cysticidal activity, corneal cytotoxicity, tissue penetration, and in vivo efficacy were not evaluated [157].

Preliminary evidence also suggests that microemulsion-based delivery may enhance the activity of poorly water-soluble compounds against *Acanthamoeba* cysts. A microemulsion containing phytosteryl glycosides isolated from *Peperomia pellucida* was reported to reduce cyst viability, with an estimated IC_50_ of 0.88 ± 0.02 µg/mL after 48 h. These findings should be interpreted cautiously because parasite identification was limited, cyst viability was assessed primarily by staining, and neither excystation capacity nor toxicity toward corneal cells was examined. Moreover, the possible contribution of the formulation components, including dimethyl sulfoxide (DMSO) and Tween 20, to the observed effect was not fully resolved [158].

In addition to nano- and microemulsion-based formulations, detachable hybrid microneedles have been investigated as a means of delivering polyhexamethylene biguanide directly into the corneal stroma. A biodegradable poly(lactic-co-glycolic acid) (PLGA) drug tip containing PHMB was evaluated in an experimental mouse model of *Acanthamoeba* keratitis. Following intracorneal insertion, the tip remained within the stromal tissue and provided sustained drug release, with approximately 93% of the model compound released during the first four days and near-complete release by day nine. A single administration of the PHMB-loaded microneedle was associated with reduced corneal opacity and lower clinical severity scores compared with untreated controls. These results provide proof of concept that localized sustained delivery may improve drug exposure at the site of infection while reducing the need for frequent topical administration [159].

Nevertheless, the microneedle study did not demonstrate complete microbiological eradication of *Acanthamoeba*, and therapeutic efficacy was evaluated primarily through corneal opacity and clinical scoring. The experimental model relied predominantly on trophozoite inoculation, the observation period was short, and the system was not directly compared with standard intensive topical PHMB treatment. The invasiveness of intracorneal administration, reproducibility of dosing, risk of additional corneal injury, drug-loading capacity, and long-term biocompatibility also require further evaluation.

Collectively, these studies support the feasibility of advanced ocular delivery platforms for anti-*Acanthamoeba* compounds, but they do not yet establish clinical efficacy. Future studies should evaluate both trophozoites and mature cysts, include clinically relevant genotypes and isolates, determine parasite burden and recurrence after treatment withdrawal, and assess toxicity in corneal epithelial cells, keratocytes, and endothelial cells. Candidate formulations should also be examined in ex vivo corneal systems and in vivo models, with direct comparison against current topical treatment regimens. For redox-modulating agents, particular attention should be given to whether controlled delivery can preserve anti-amoebic activity while limiting oxidative or nitrosative injury to host corneal tissues [157,158,159].

### 6.2. Optimization and Standardization of Photodynamic Therapies

Future development of photodynamic therapies for *Acanthamoeba* keratitis should prioritize the definition of a clinically relevant therapeutic window. Experimental protocols should standardize photosensitizer concentration, incubation time, irradiation wavelength, fluence rate, total light dose, oxygen availability, and treatment depth. Both trophozoites and mature cysts should be evaluated using culture-confirmed viability or excystation assays rather than metabolic staining alone. Studies should also include multiple clinical isolates and directly compare anti-amoebic activity with toxicity toward corneal epithelial cells, keratocytes, and endothelial cells.

The divergent findings obtained with hypocrellin B, the cationic phthalocyanine RLP068, and methylene blue demonstrate that photodynamic efficacy is determined not simply by reactive-species generation, but also by photosensitizer localization and retention, accessibility of cellular targets, parasite stage, and resistance of the cyst wall [139,140,142]. Ex vivo corneal models and in vivo studies are therefore required to determine tissue penetration, stromal drug distribution, epithelial safety, treatment-associated inflammation, and recurrence after therapy. Combination protocols involving photodynamic therapy and conventional anti-amoebic agents should also be investigated. In particular, methylene blue-mediated photodynamic therapy produced enhanced combined activity with polyhexamethylene biguanide and amphotericin B, but not voriconazole; formal interaction analyses are required to establish whether these effects represent true pharmacodynamic synergy and whether they could support dose reduction [142].

Oxygen availability should be treated as a controlled experimental variable rather than merely a descriptive parameter in photoactivated corneal therapies. During riboflavin/UVA cross-linking, dissolved stromal oxygen is rapidly depleted within seconds of irradiation and is replenished when irradiation is interrupted [160]. Moreover, in porcine corneas, CXL performed under a low-oxygen atmosphere (<0.1% O_2_) produced substantially less biomechanical stiffening than the same protocol performed under normal atmospheric oxygen conditions [161]. Although these studies were not conducted in infected corneas, they demonstrate that oxygenation can materially influence CXL photochemistry and treatment outcome. Oxygen conditions should therefore be reported and standardized in experimental PACK-CXL protocols for AK.

Conflicting experimental findings also highlight the need to standardize post-irradiation conditions and outcome assessment. Future studies should define the duration of chromophore exposure before and after irradiation, chromophore washout procedures, control of ambient-light exposure, and the time points used for viability assessment. This is particularly important because prolonged retention of riboflavin in culture medium may produce continuing chemical or photochemical effects that do not reflect its pharmacokinetics in the cornea [147]. For cysts, membrane-integrity staining should be complemented by long-term excystation and regrowth assays to distinguish transient membrane injury from irreversible cyst death.

Fluence optimization should also consider both direct parasite inactivation and protection of corneal tissue. Higher-fluence riboflavin/UVA and rose bengal/green-light protocols can increase stromal resistance to collagenase digestion, but this non-amoebicidal benefit must be experimentally separated from trophocidal or cysticidal activity [152]. Future studies should therefore report parasite survival, stromal resistance to proteolysis, treatment depth, and corneal-cell toxicity as distinct outcomes rather than treating clinical stabilization as evidence of microbiological eradication.

### 6.3. Targeted Modulation of Innate Immune Recruitment

Single-cell RNA sequencing of corneal tissues from patients with advanced infectious keratitis indicated a markedly lower representation of neutrophils in *Acanthamoeba* keratitis than in bacterial or fungal keratitis. Cell–cell communication analysis additionally suggested attenuation of signaling associated with C-X-C motif chemokine ligands (CXCLs), C-C motif chemokine ligands (CCLs), and tumor necrosis factor (TNF), including pathways involved in neutrophil chemotaxis [9]. These findings support reduced chemokine-mediated neutrophil recruitment as a possible contributor to persistent AK, although the analysis included only three AK corneas obtained during therapeutic keratoplasty and therefore reflected late-stage disease.

In the experimental AK model, CXCL1 supplementation reduced the corneal *Acanthamoeba* burden, but its effects were strongly dose-dependent. Low-dose CXCL1 reduced the detectable burden without significantly improving clinical scores, whereas high-dose CXCL1 produced a greater reduction, approaching zero by day 7, but was accompanied by severe corneal dissolution approaching perforation [9]. These findings indicate that generalized enhancement of neutrophil recruitment may improve parasite control while simultaneously intensifying tissue injury.

To improve localization of chemokine activity, Wei et al. [9] developed the *Acanthamoeba*-binding Acab95–CXCL1 fusion protein. The construct retained parasite-binding and neutrophil-chemotactic activity and produced slower, more sustained recruitment than unconjugated CXCL1, with neutrophils concentrated around cyst-containing regions. By day 7, treated corneas showed clinical improvement and no detectable *Acanthamoeba* using in vivo confocal microscopy, quantitative polymerase chain reaction (qPCR), or histological examination. However, the short follow-up, intrastromal administration, small experimental groups, predominant use of a single clinical T4 isolate, and absence of culture-based regrowth or long-term excystation testing preclude conclusions regarding permanent cyst eradication or recurrence.

Future studies should determine which neutrophil effector mechanisms—including NOX2-derived ROS, MPO-associated oxidants, proteases, and NET-associated components—are responsible for parasite clearance and which primarily contribute to stromal injury [7,8,9,86]. Targeted chemokine delivery should also be evaluated together with conventional anti-amoebic treatment, across multiple clinical isolates and mature cyst models, using longer post-treatment follow-up and less invasive ocular delivery systems.

### 6.4. Experimental Priorities for Redox Research in Acanthamoeba Keratitis

Future redox research in *Acanthamoeba* keratitis should move beyond descriptive associations and determine whether oxidative and nitrosative processes are causally involved in parasite killing, host-tissue injury, or both. Increased oxidant-associated fluorescence or altered antioxidant-enzyme expression should not automatically be interpreted as evidence that redox imbalance is the primary mechanism of parasite death. Future studies should combine time-resolved redox measurements with functional interventions that inhibit, enhance, or selectively disrupt the pathway under investigation. Because commonly used probes such as DCFDA do not identify the reactive species involved or their intracellular source, additional assays are required. These should include measurements of superoxide, hydrogen peroxide, nitric oxide and other RNS, lipid and protein oxidation, glutathione redox status, mitochondrial membrane potential, and ATP production. Establishing whether redox disruption precedes or follows loss of viability will be essential for distinguishing a primary killing mechanism from a secondary consequence of cellular injury [10,12,122,123,124].

Parasite viability must also be defined using stage-specific and biologically meaningful endpoints. Reduced metabolic activity, loss of motility, altered morphology, or transient membrane permeability should not be equated with irreversible killing, particularly in mature cysts. Trophozoites and cysts should therefore be assessed separately, and treatment should be followed by compound washout, long-term excystation, and regrowth assays. Studies should include multiple clinical isolates and, where feasible, representatives of different genotypes, because findings obtained with a single laboratory strain may not be broadly generalizable. Experimental concentrations, exposure times, and dosing schedules should reflect clinically achievable ocular-surface residence time and corneal penetration. After initial screening in parasite monocultures, promising interventions should progress to corneal-cell cocultures, ex vivo corneal systems, and appropriately designed in vivo models [5,144,145].

Validation of parasite redox targets requires evidence extending beyond stress-induced expression or inhibition of an isolated recombinant enzyme. Candidate targets should be evaluated through gene silencing or other loss-of-function approaches, selective pharmacological inhibition, and, where feasible, rescue experiments. Intracellular target engagement should be demonstrated in intact trophozoites and cysts, because biochemical inhibition does not establish that the same target is reached under cellular conditions. The thioredoxin system illustrates this challenge: the small and large thioredoxin reductases are biochemically inhibitable, but enzyme inhibition and trophozoite killing do not correlate consistently, while activation of glutathione-dependent pathways may compensate for thioredoxin-system disruption. Target essentiality, intracellular accessibility, and compensatory redox responses should therefore be established before an antioxidant enzyme is considered a validated therapeutic vulnerability [11,21,117].

Anti-*Acanthamoeba* activity and host-cell selectivity should be evaluated in parallel. A therapeutically relevant redox intervention must affect trophozoites and mature cysts at concentrations that preserve corneal epithelial cells, stromal keratocytes, and, where relevant, endothelial cells. Host-cell assessment should include viability, barrier integrity, wound healing, mitochondrial function, apoptosis, and inflammatory signaling. Host-directed antioxidants require additional scrutiny because protection of corneal tissue may be accompanied by suppression of oxidant-dependent innate immune mechanisms or attenuation of therapies that rely on ROS or RNS generation. Combination studies should therefore assess not only whether two agents are additive or synergistic, but also their sequence, timing, and spatial distribution. An antioxidant may be beneficial after substantial reduction in the parasite burden but detrimental during the early phase of pro-oxidant or immune-mediated parasite clearance. Conversely, complementary oxidative and nitrosative systems may enhance parasite killing but narrow the therapeutic window [9,86,127,128,132].

Finally, future studies should determine whether sublethal redox stress promotes encystment, persistent antioxidant adaptation, altered excystation, or reduced susceptibility to repeated treatment. Serial-exposure experiments combined with delayed regrowth, virulence assessment, and post-treatment transcriptomic or proteomic analysis could distinguish transient stress responses from stable adaptive phenotypes [155,156]. Standardized reporting should include parasite species or genotype, isolate origin, developmental stage, inoculum size, culture conditions, treatment concentration and duration, compound removal or neutralization, redox endpoints, host-cell toxicity, and long-term excystation or regrowth. Such standardization is essential for comparing studies and identifying redox-based strategies with genuine translational potential.

## 7. Conclusions

*Acanthamoeba* keratitis provides a clinically relevant model of redox conflict at the host–parasite interface. Host-derived ROS and RNS may contribute to parasite control through neutrophil- and macrophage-associated mechanisms, yet excessive or poorly localized oxidative responses can simultaneously aggravate stromal inflammation and corneal tissue injury. *Acanthamoeba*, in turn, possesses a broad and adaptable antioxidant network involving SOD, CAT, thioredoxin- and glutathione-dependent pathways, peroxiredoxins, and mitochondrial mechanisms that may support survival under inflammatory, environmental, and pharmacological stress. However, the relative contribution of these pathways to trophozoite persistence, cyst resistance, and disease progression in the infected cornea remains incompletely defined.

These observations identify parasite redox homeostasis as a potential therapeutic vulnerability but also illustrate the limitations of non-selective redox modulation. Direct inhibition of antioxidant enzymes, disruption of mitochondrial and thiol metabolism, and controlled ROS- or RNS-generating approaches may enhance parasite killing, whereas host-directed antioxidants may limit secondary corneal injury. Nevertheless, neither indiscriminate amplification nor broad suppression of reactive species is likely to provide an optimal therapeutic strategy. Excessive pro-oxidant activity may damage epithelial cells, keratocytes, and endothelial cells, while excessive antioxidant exposure may weaken oxidant-dependent innate immune mechanisms or interfere with redox-based antimicrobial therapies. Effective treatment will therefore require spatially, temporally, and stage-specifically controlled modulation that weakens parasite defenses while preserving corneal integrity and essential host responses.

Current evidence remains largely preclinical and is frequently based on single laboratory strains, trophozoite-dominated models, indirect redox measurements, and short-term viability assays. Future studies should establish causal links between specific redox pathways and parasite survival, evaluate multiple clinical isolates and genotypes, distinguish trophocidal from cysticidal activity, and confirm irreversible cyst inactivation through long-term excystation and regrowth assays. Parallel assessment of corneal-cell toxicity, immune function, tissue penetration, and clinically achievable exposure will be essential for defining a genuine therapeutic window. Integrating parasite-directed redox disruption with targeted delivery, conventional anti-amoebic treatment, and controlled protection of host tissues may ultimately provide a more rational approach to this environmentally acquired, sight-threatening infection.

## Data Availability

No new data were created or analyzed in this study. The original illustrative Figure 1 is publicly available in Zenodo at https://doi.org/10.5281/zenodo.21864012. The corresponding raw micrographs are available from the corresponding authors upon reasonable request.

## Supplementary Materials

The following supporting information can be downloaded at: https://www.mdpi.com/article/10.3390/ijms27167293/s1.

## Abbreviations

The following abbreviations are used in this manuscript:

3-AT	3-amino-1,2,4-triazole
AcCP3	*Acanthamoeba castellanii* cysteine protease 3
AcCS	*Acanthamoeba castellanii* cysteine synthase
AcFe-SOD	iron-containing superoxide dismutase of *Acanthamoeba castellanii*
AcTrx1	*Acanthamoeba castellanii* thioredoxin 1
AcTrxR-L	large vertebrate-type thioredoxin reductase of *Acanthamoeba castellanii*
AcTrxR-S	small bacterial-type thioredoxin reductase of *Acanthamoeba castellanii*
AcUCP	*Acanthamoeba castellanii* uncoupling protein
AK	*Acanthamoeba* keratitis
AP	apurinic/apyrimidinic
ATCC	American Type Culture Collection
ATP	adenosine triphosphate
Bax	Bcl-2-associated X protein
Bcl-2	B-cell lymphoma 2
CAT	catalase
CCAP	Culture Collection of Algae and Protozoa
CCL	C-C motif chemokine ligand
CEC	corneal epithelial cell
COX	cyclooxygenase
cPLA2α	cytosolic phospholipase A2 alpha
CXCL	C-X-C motif chemokine ligand
CXL	corneal cross-linking
DALK	deep anterior lamellar keratoplasty
DCFDA	2′,7′-dichlorodihydrofluorescein diacetate
DMSO	dimethyl sulfoxide
EU	European Union
GLA	glabridin
GPx	glutathione peroxidase
GR	glutathione reductase
GSH	reduced glutathione
HCEC	human corneal epithelial cell
HET-CAM	hen’s egg test on the chorioallantoic membrane
HO-1	heme oxygenase 1
IC_50_	half-maximal inhibitory concentration
IL	interleukin
ISL	isoliquiritigenin
KCN	potassium cyanide
LK	lamellar keratoplasty
MB	methylene blue
MBP	mannose-binding protein
MDA	malondialdehyde
MIP-133	mannose-induced protease 133
MMP	matrix metalloproteinase
MPO	myeloperoxidase
mRNA	messenger RNA
MT2	melatonin receptor 2
MyD88	myeloid differentiation primary response 88
NAC	N-acetylcysteine
NAD^+^	oxidized nicotinamide adenine dinucleotide
NADH	reduced nicotinamide adenine dinucleotide
NADH-FRD	NADH-dependent fumarate reductase
NADPH	reduced nicotinamide adenine dinucleotide phosphate
NET	neutrophil extracellular trap
NF-κB	nuclear factor kappa B
NO	nitric oxide
NOX2	NADPH oxidase 2
NOX4	NADPH oxidase 4
Nrf2	nuclear factor erythroid 2-related factor 2
ODAK	Orphan Drug for *Acanthamoeba* Keratitis
ONOO^−^	peroxynitrite
PACT	photodynamic antimicrobial chemotherapy
PACK-CXL	photoactivated chromophore for keratitis–corneal cross-linking
PAMP	pathogen-associated molecular pattern
PDT	photodynamic therapy
PHMB	polyhexamethylene biguanide
PINK1	PTEN-induced kinase 1
PKP	penetrating keratoplasty
PLGA	poly(lactic-co-glycolic acid)
Prx	peroxiredoxin
Prx-1	peroxiredoxin 1
qPCR	quantitative polymerase chain reaction
RNS	reactive nitrogen species
ROS	reactive oxygen species
SDH	succinate dehydrogenase
SERPINA3K	serpin family A member 3K
SIRC	Statens Seruminstitut rabbit corneal cell line
SOD	superoxide dismutase
STAT3	signal transducer and activator of transcription 3
TLR	Toll-like receptor
TNF	tumor necrosis factor
Trx	thioredoxin
Trx-1	thioredoxin 1
TrxR	thioredoxin reductase
TRi-1 and TRi-2	thioredoxin reductase inhibitors 1 and 2
UVA	ultraviolet A
